# Photoactivatable CaMKII induces synaptic plasticity in single synapses

**DOI:** 10.1038/s41467-021-21025-6

**Published:** 2021-02-02

**Authors:** Akihiro C. E. Shibata, Hiromi H. Ueda, Kei Eto, Maki Onda, Aiko Sato, Tatsuko Ohba, Junichi Nabekura, Hideji Murakoshi

**Affiliations:** 1 Supportive Center for Brain Research, National Institute for Physiological Sciences, Okazaki, Aichi 444-8585 Japan; 2 Department of Physiological Sciences, The Graduate University for Advanced Studies, Hayama, Kanagawa 240-0193 Japan; 3 Division of Homeostatic Development, National Institute for Physiological Sciences, Okazaki, Aichi 444-8585 Japan

**Keywords:** Optogenetics, Long-term potentiation

## Abstract

Optogenetic approaches for studying neuronal functions have proven their utility in the neurosciences. However, optogenetic tools capable of inducing synaptic plasticity at the level of single synapses have been lacking. Here, we engineered a photoactivatable (pa)CaMKII by fusing a light-sensitive domain, LOV2, to CaMKIIα. Blue light or two-photon excitation reversibly activated paCaMKII. Activation in single spines was sufficient to induce structural long-term potentiation (sLTP) in vitro and in vivo. paCaMKII activation was also sufficient for the recruitment of AMPA receptors and functional LTP in single spines. By combining paCaMKII with protein activity imaging by 2-photon FLIM-FRET, we demonstrate that paCaMKII activation in clustered spines induces robust sLTP via a mechanism that involves the actin-regulatory small GTPase, Cdc42. This optogenetic tool for dissecting the function of CaMKII activation (i.e., the sufficiency of CaMKII rather than necessity) and for manipulating synaptic plasticity will find many applications in neuroscience and other fields.

## Abstract (web summary)

Optogenetic control of molecules is important in cell biology and neuroscience. Here, the authors describe an optogenetic tool to control the Ca^²+^/calmodulin-dependent protein kinase II and use it to control plasticity at the single synapse level.

## Introduction

Long-term potentiation (LTP) at excitatory synapses is thought to be the basis of learning and memory. Excitatory synapses consist of presynaptic terminals and postsynaptic membranes, and the postsynaptic components (i.e., postsynaptic density, PSD) typically reside in protrusions called dendritic spines. Postsynaptic receptors detect chemical transmitters, such as glutamate, which are released from presynapses and can lead to LTP. The events underlying LTP are well studied in hippocampal slices. Released glutamate binds to N-methyl-D-aspartate (NMDA)-type glutamate receptors (NMDARs) in the postsynaptic membrane, allowing Ca^2+^ influx through the receptor, which activates various signaling proteins and changes the protein composition in the spines^1^. These events cause long-lasting spine enlargement called structural (s)LTP^2^ and functional LTP associated with accumulation of α-amino-3-hydroxy-5-methyl-4-isoxazole propionic acid (AMPA)-type glutamate receptors (AMPARs) in the PSD^3,4^.

Ca^2+^/calmodulin-dependent protein kinase II (CaMKII) is an essential signaling protein for LTP induction^5–8^. CaMKII is a serine/threonine protein kinase that is abundant in cortical and hippocampal neurons, and consists of 12–14 α and β subunits at a ratio of 3:1^9,10^. They are activated by Ca^2+^/calmodulin binding, leading to a conformational change and activation of CaMKII^8,11^. Activated kinase autophosphorylates threonine residue 286 (T286) in the regulatory domain of neighbor subunits^8,11^. Two-photon excitation-based imaging studies revealed that glutamate stimulation recruits CaMKII into stimulated spines^12,13^, and the activation is spine specific^14,15^. The importance of CaMKII function in synaptic plasticity has been extensively studied, mostly using loss-of-function assays with inhibitory drugs, peptides, or gene silencing^5–8,16^. A few studies using purified CaMKII found that introducing active CaMKII into the cytosol of a neuron is sufficient to trigger LTP^17–19^. However, the direct effect of CaMKII activation in single spines has remained elusive because of the lack of suitable tools.

Recently, numerous genetically encoded photoactivatable signaling proteins have been developed and used to study cellular functions^20^, such as synaptic functions^21–26^. These optogenetic tools are revolutionizing neuroscience and, more broadly, molecular cell signaling studies. Here, we developed a photoactivatable CaMKII (paCaMKII) that fuses CaMKII and the light-oxygen-voltage domain 2 (LOV2)-Jα of the plant photoreceptor, phototropin 1^27^. By expressing this optogenetic paCaMKII in hippocampal and cortical neurons, we demonstrate that paCaMKII activation is sufficient to induce sLTP, recruit AMPARs into dendritic spines, and induce functional LTP at the single-spine level. Moreover, by combining paCaMKII with two-photon fluorescence lifetime imaging microscopy-based Förster resonance energy transfer (2pFLIM-FRET) to image protein activity, we show that paCaMKII activation in a single spine robustly activates the actin-regulatory small GTPase Cdc42 rather than RhoA. paCaMKII activation in clustered spines enhances Cdc42 activity, and subsequent sLTP likely contributes to the molecular mechanism of clustered synaptic plasticity^28^. Thus, paCaMKII is a significant addition to the current optogenetic toolbox, and allows manipulation of synaptic plasticity and neuronal cell signaling.

## Results

### Development of a paCaMKII

We decided to use LOV2-Jα rather than other light-sensing protein domains to create a paCaMKII because of the following reasons. First, LOV2-Jα acts with single component^27^, whereas other available light sensors, such as CRY2-CIBN^22^, Dronpa^29^, PhyB^30^, and UVR8^31^, require the multiple components. Second, LOV2-Jα is ~140 amino acids (a.a.), which is smaller than most other sensors (i.e., CRY2/CIBN 498/170 a.a., Dronpa 257 a.a., PhyB 908 a.a., and UVR8 124 a.a.). Furthermore, to our knowledge, while the two-photon cross sections of Dronpa, PhyB, and UVR8 have not been determined, it is known that the light-absorbing cofactor of LOV2, flavin mononucleotide (FMN), has a relatively large two-photon cross section (0.5–0.9 GM in 800–900 nm)^32^. It is larger than that of flavin adenine dinucleotide, the light-absorbing cofactor for CRY2 (0.02–0.04 GM in 800–900 nm)^33^. Finally, the LOV2 works in a reversible manner (~40 s), similar to the others.

**Fig. 1:**
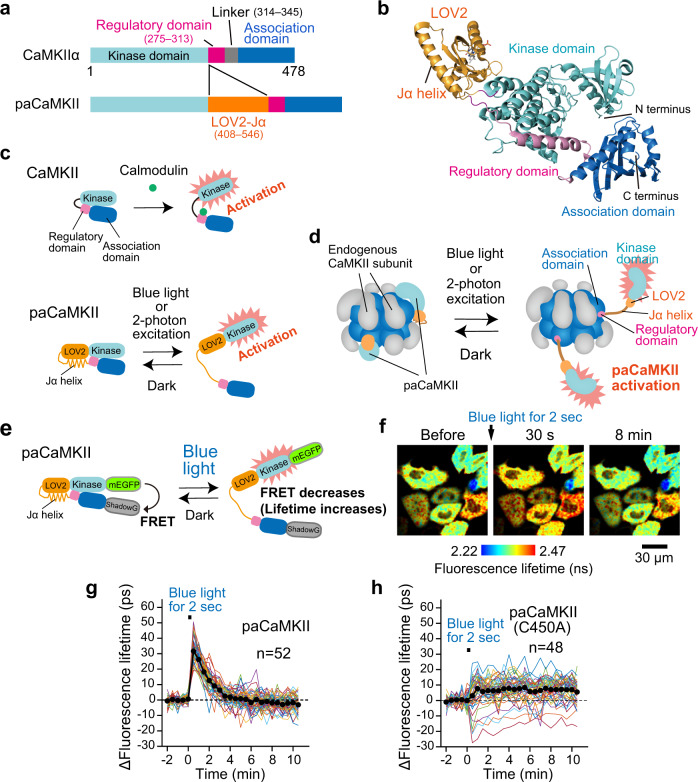
Development of paCaMKII. **a** CaMKIIα and paCaMKII domain structures. **b** Structural model of paCaMKII, visualized with PyMOL. The LOV2, kinase, regulatory, and association domains are shown as yellow-, cyan-, magenta-, blue-colored cartoons, respectively. Note that this structure is putative, and was created by available structural data (PDB: #3SOA and 2V1B)^34,35^. **c** Schematic drawing of endogenous and paCaMKII activation. **d** Schematic drawing of paCaMKII activation in the oligomeric state. Note that most CaMKII and paCaMKII likely exist in oligomeric form in cells. **e** A schematic of the conformational change of the paCaMKII FRET construct. mEGFP and ShadowG^66^ were fused to the N- and C-terminus, respectively. Blue light illumination induces the structural change of paCaMKII (i.e., the distance between mEGFP and ShadowG becomes longer), leading to decreased FRET and increased mEGFP fluorescence lifetime. **f** Representative fluorescence lifetime images of mEGFP-paCaMKII-ShadowG after blue LED light illumination for 2 s at 35 mW cm^−2^. For imaging, two-photon excitation at 920 nm was used to excite mEGFP. The lifetime change (conformational change) of mEGFP-paCaMKII-ShadowG (**g**) and its light-insensitive mutant (C450A) (**h**) in individual HeLa cells after blue light illumination. Colored lines represent the response signal from individual cells, and the black circles indicate the average time course. The data are presented as mean ± SEM. The number of samples (*n*) is indicated in the respective panels. Source data are provided as a Source Data file.

To develop a photoactivatable (pa)CaMKII, we first looked at the crystal structures of CaMKIIα (PDB: #3SOA)^34^ and LOV2-Jα (PDB: #2V1B)^35^, and decided to insert the LOV2-Jα sequence into the hinge region of rat CaMKIIα (Fig. 1a, Supplementary Fig. 1). To compensate LOV2 insertion, we deleted a flexible linker region (315–344) which affects the configuration and balance between active and inactive state^10,34,36^, expecting that paCaMKII forms a similar configuration with endogenous CaMKII. We also expected that closed paCaMKII conformation in the dark would cause the regulatory domain to inhibit kinase activity, while light illumination would release the regulatory domain from the kinase domain, thereby activating paCaMKII (Fig. 1b–d).

We created various light-sensitive CaMKII “prototypes” by varying the linker and LOV2 insertion positions in the CaMKII fusion protein. We tested the prototypes for light-dependent conformational changes by expressing them in HeLa cells. Then, we evaluated photoactivation using 2pFLIM-FRET (Fig. 1e–h, Supplementary Fig. 2)^37^. We also biochemically validated the prototypes in HeLa cells by assaying light-dependent autophosphorylation at T286 (Supplementary Fig. 3). Among them, the construct with the LOV2-Jα sequence (residues 408–546) inserted into the hinge region of CaMKIIα between residues 275 and 278 exhibited light-dependent conformational changes and autophosphorylation (Supplementary Figs. 1, 2a, i, and 3a, b). The fluorescence lifetime change of mEGFP-paCaMKII-ShadowG in individual cells upon light illumination was variable ranging from 10 to 60 ps (Supplementary Fig. 2a, i). Next, we introduced four previously reported mutations (F394L/I419V/A430T/I434T)^38^ into the association domain (Supplementary Fig. 1). These mutations minimize the response variability and improve the aggregation of the CaMKII FRET sensor, but do not affect the formation of oligomer^38^. The presence of these four mutations reduced light-dependent cell-to-cell variability (Supplementary Fig. 2b, i; *F*-test, *p* = 2.675 × 10^−5^, compared to the prototype) and suppressed kinase activity in both dark/light conditions (Supplementary Fig. 3b). Also, introducing the four mutations increased the basal fluorescence lifetime (Supplementary Fig. 2h). This increase could be due to improved aggregation, as seen previously^38^. Finally, we introduced the S279K mutation into the regulatory domain. This mutation improved the dynamic range of autophosphorylation (Supplementary Fig. 3b) while reducing light-dependent cell-to-cell response variability (Supplementary Fig. 2a, c, i; *F*-test, *p* = 0.0168, compared to the prototype). We named this mutant (the prototype with the five mutations) as paCaMKII and used for further studies.

**Fig. 2:**
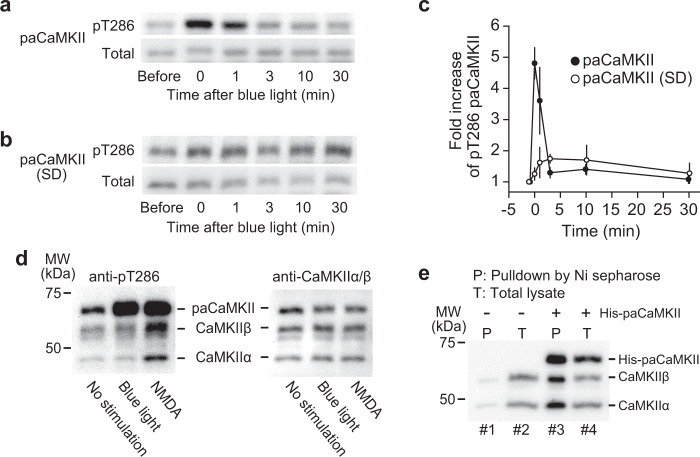
Light-dependent paCaMKII autophosphorylation. The time courses of paCaMKII (**a**) and light-insensitive super-dark paCaMKII (SD) mutant (**b**) dephosphorylation at T286 after light illumination. Dissociated hippocampal neurons were infected with AAV-DJ encoding Flag-His×6-paCaMKII or mutant under CaMKII promotor were illuminated with blue light for 3 min. Right after illumination (at 0 min in the figure), samples were incubated in the dark for the indicated time. **c** Quantification of **a** and **b**. The number of samples (*n*, biologically independent) at each point is as the following. For paCaMKII, before, *n* = 8; 0 min, *n* = 8; 1 min *n* = 4; 3 min, *n* = 4; 10 min, *n* = 4; 30 min, *n* = 8. For paCaMKII(SD), before, *n* = 6; 0 min, *n* = 6; 1 min *n* = 4; 3 min, *n* = 3; 10 min, *n* = 4; 30 min, *n* = 6. The band intensity of before light was used for normalization. The band intensities of pT286 were divided by those of total. The data are presented as mean ± SEM. **d** Selectivity of paCaMKII. Dissociated hippocampal neurons were infected with AAV-DJ encoding Flag-His×6-paCaMKII under CaMKII promotor were stimulated with no light (lane 1), blue light for 5 min (lane 2), or 20 µM NMDA for 2 min (lane 3). The left panel shows the autophosphorylation of paCaMKII and CaMKIIα/β, and the right panel shows the expression of the respective proteins. Light illumination selectively activates paCaMKII, but not CaMKIIα/β (lane 2 in the left panel), whereas NMDA stimulation activates paCaMKII and CaMKIIα/β (lane 3 in the left panel). **e** Incorporation of paCaMKII into CaMKIIα/β oligomer. Dissociated cortical neurons with no transfection (lanes 1 and 2) and AAV-DJ encoding Flag-His×6-paCaMKII transfection (lanes 3 and 4) were lysed and pulled down with Ni sepharose followed by immunoblotting with CaMKIIα/β antibody. In lane 3, Flag-His×6-paCaMKII was pulled down, and subsequent western blotting identified that CaMKIIα/β are incorporated, suggesting that paCaMKII, CaMKIIα, and CaMKIIβ form oligomer. Source data are provided as a Source Data file.

The conformational change in paCaMKII occurs soon after a light pulse and recovers within a few minutes (Supplementary Fig. 2c). In cultured neurons, paCaMKII autophosphorylated upon light illumination and were dephosphorylated within a few minutes in the dark (Fig. 2a, c). Autophosphorylation was not observed with a light-insensitive paCaMKII(SD) mutant, in which LOV2 was replaced with a light-insensitive super-dark LOV2(SD) mutant (Fig. 2b, c)^39^. An autophosphorylation-deficient mutant (T286A) and a kinase-dead mutant (K42M) did not exhibit autophosphorylation (Supplementary Fig. 3a), while the introduction of the mutations did not prevent a light-dependent conformational change (Supplementary Fig. 2d, e). The T286A/K42M mutants exhibited a slight decrease in the conformational change. It is probably due to the lack of autophosphorylation (Supplementary Fig. 2i). Introducing a constitutively open-form I539E mutation^27^ in the Jα helix induced a relatively high basal fluorescence lifetime, most likely due to the open form of the mutant (Supplementary Fig. 2h). Also, this I539E mutant exhibited a slight light-dependent structural change (Supplementary Fig. 2f, i), implying the existence of another contact site between CaMKII and LOV2 in addition to the LOV2 and Jα helix interaction. In contrast, introducing a light-insensitive C450A mutation caused only a small and irreversible change after light illumination (Supplementary Fig. 2g, i).

Next, to test whether light selectively activates paCaMKII, we measured the autophosphorylation of paCaMKII, CaMKIIα, and CaMKIIβ upon blue light illumination. We observed that the light selectively induced the autophosphorylation of paCaMKII, but not CaMKIIα or CaMKIIβ (Fig. 2d). In contrast, NMDA stimulation induced the autophosphorylation of paCaMKII/CaMKIIα/CaMKIIβ in the dark, suggesting that an NMDA receptor-dependent Ca^2+^ increase activates paCaMKII, similar to the endogenous form (Fig. 2d). In addition, pull-down assay with His-tagged paCaMKII confirmed that paCaMKII and CaMKIIα/β interact (Fig. 2e), indicating that paCaMKII and CaMKIIα/β form oligomers similar to endogenous CaMKII subunits.

### Two-photon paCaMKII activation in single dendritic spines triggers sLTP

**Fig. 3:**
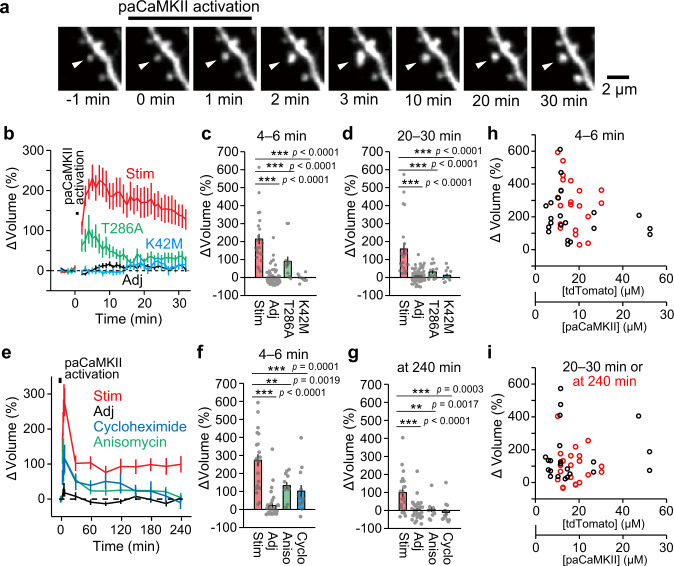
paCaMKII activation triggers structural plasticity. **a** Two-photon fluorescence images of dendritic spines during the induction of sLTP by two-photon paCaMKII activation. A hippocampal CA1 neuron expressing tdTomato-P2A-paCaMKII was observed by two-photon excitation at 1000 nm, and paCaMKII was activated at 900 nm in a spine indicated by white arrows. **b** Averaged time courses of spine volume change in the stimulated spine (Stim) and adjacent spines (2–10 µm, Adj). Data using paCaMKII mutants (T286A and K42M) are also shown. *n*(spines/neurons) = 23/11 stimulated spines, 67/11 adjacent spines, 13/6 T286A, and 11/5 K42M. Quantification of transient (**c**, averaged over 4–6 min) and sustained (**d**, averaged over 20–30 min) spine volume change. *n*(spines/neurons) is the same as in **b**. **e** Averaged time courses of long-term observation of spine volume change of the stimulated spine (Stim) and adjacent spines (Adj). Data using protein synthesis inhibitors (50 µM cycloheximide and 50 µM anisomycin) are also shown. *n*(spines/neurons) = 21/8 stimulated spines, 33/8 adjacent spines, 12/4 cycloheximide, and 12/4 anisomycin. Quantification of transient (**f**, averaged over 4–6 min) and sustained spine volume change (**g**, at 240 min). *n*(spines/neurons) is the same as in **e**. **h**, **i** The dependency of the paCaMKII expression level on spine enlargement following two-photon excitation. The data points of Stim used in **c** and **f** were replotted against tdTomato and paCaMKII concentration with black and red circles in **h**, respectively. The data points of Stim used in **d** and **g** were replotted with black and red circles in **i**, respectively. The tdTomato expression was measured by comparing the fluorescence intensity of tdTomato in proximal dendrites and a known concentration of purified tdTomato under a two-photon microscope. Because the ratio of tdTomato and paCaMKII expression is 2:1 (see Supplementary Fig. 4), the scale for paCaMKII concentration is presented as well. For **b**–**g**, the data are presented as mean ± SEM. Statistical comparisons were performed using one-way ANOVA followed by Dunnett’s post-hoc test. ***p < 0.001; **p < 0.01; *p < 0.05; N.S. not significant. Source data are provided as a Source Data file.

To test whether paCaMKII activation induces sLTP in single dendritic spines, we measured the change in spine volume in response to paCaMKII activation in single spines (Fig. 3a, b). We transfected CA1 pyramidal neurons in cultured hippocampal slices using biolistic gene transfer with a vector encoding paCaMKII and the red fluorescent protein tdTomato (tdTomato-P2A-paCaMKII). We monitored tdTomato red fluorescence by a two-photon microscope as a measure of spine volume change. To induce paCaMKII activation in a single dendritic spine, we applied a low-frequency train of two-photon excitation pulses to the spine (900 nm, 30 pulses, 1 Hz, 80 ms duration/pulse, 4 mW). paCaMKII activation rapidly increased spine volume by ~220% (Fig. 3b, c) and relaxed to an elevated level (150%) after 20–30 min (Fig. 3b, d). Upon paCaMKII activation, we observed a slight increase in adjacent spine volume (Fig. 3b). This might be due to paCaMKII cross-photoactivation by the 1000 nm imaging laser during observation. Activation of the K42M kinase-dead mutant did not change spine volume (Fig. 3b–d). Activation of the T286A autophosphorylation-deficient mutant induced a smaller and transient volume change compared with that of paCaMKII activation (Fig. 3b–d). These data suggest that spine enlargement is due to the light-induced increase of kinase activity and autophosphorylation of paCaMKII.

Long-term observation of dendritic spines following paCaMKII activation in hippocampal slices revealed that paCaMKII-induced sLTP persists for over 4 h (Fig. 3e). Since previous studies suggested that glutamate-induced sLTP depends on protein synthesis in some conditions^40,41^, we tested whether the paCaMKII-dependent sLTP depends on protein synthesis. Two different protein synthesis inhibitors, anisomycin or cycloheximide, reduced the transient volume change and inhibited persistent sLTP (Fig. 3e–g), suggesting that long-lasting sLTP requires de novo protein synthesis.

In addition, we confirmed that paCaMKII trigger sLTP in a broad concentration range (3–25 µM) (Fig. 3h, i).

### paCaMKII activation induces sLTP without increasing Ca^2+^

**Fig. 4:**
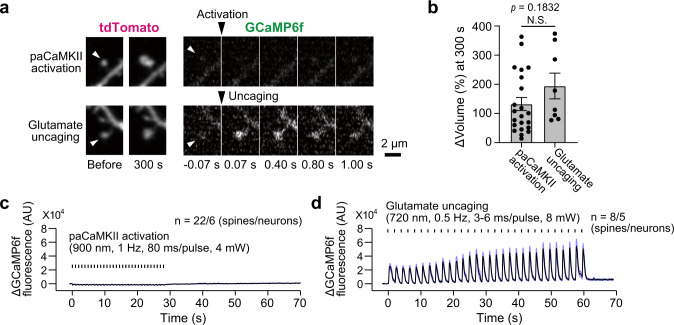
Calcium imaging during glutamate uncaging and paCaMKII activation. **a** Images of dendritic spines of a neuron expressing Ca^2+^ indicator GCaMP6f and tdTomato-P2A-paCaMKII. Both GCaMP6f and tdTomato were simultaneously imaged by two-photon excitation at 1000 nm. paCaMKII activation at 900 nm (top) or glutamate uncaging at 720 nm (bottom) was used to stimulate the spine, respectively. Images were slightly noisy because they were acquired at a high temporal resolution (15.6 Hz). GCaMP6f and tdTomato images were averaged over 3 and 30 frames, respectively. Arrowheads indicate the stimulated spines. **b** Quantification of spine volume change at 300 s. *n*(spines/neurons) = 22/6-paCaMKII activation, 8/5 glutamate uncaging. The data are presented as mean ± SEM; N.S. not significant, represents *p* > 0.05; two-tailed unpaired *t* test. Ca^2+^ transients in the stimulated spine by paCaMKII uncaging (**c**) or glutamate uncaging (**d**). The traces were averaged by three frames moving average. The number of samples is the same in **b**, and also indicated in the figures. Source data are provided as a Source Data file.

CaMKII is associated with L-, T-, and P/Q-type voltage-gated calcium channels^42–44^. Since these channels may localize in the PSD, these channels may alter Ca^2+^ dynamics in spines upon paCaMKII activation. To examine whether paCaMKII activation in single spines increases Ca^2+^ in spines, we cotransfected pyramidal neurons biolistically with expression vectors encoding tdTomato-P2A-paCaMKII and the Ca^2+^ indicator GCaMP6f^45^. We used two-photon microscopy to measure the GCaMP6f fluorescence in dendritic spines. We found that paCaMKII activation in single spines induced no detectable Ca^2+^ increase, but increased spine volume (Fig. 4a–c). In contrast, photorelease of glutamate from the caged neurotransmitter 4-methoxy-7-nitroindolinyl (MNI)-glutamate increased both spine volume and Ca^2+^ concentration in the same set of neurons used in paCaMKII experiment (Fig. 4a, b, d), indicating that GCaMP6f was functional in these neurons. Thus, we concluded that optogenetic paCaMKII activation induces sLTP without increasing Ca^2+^ levels in spines.

### Activation of paCaMKII induces AMPA receptor recruitment and functional LTP

**Fig. 5:**
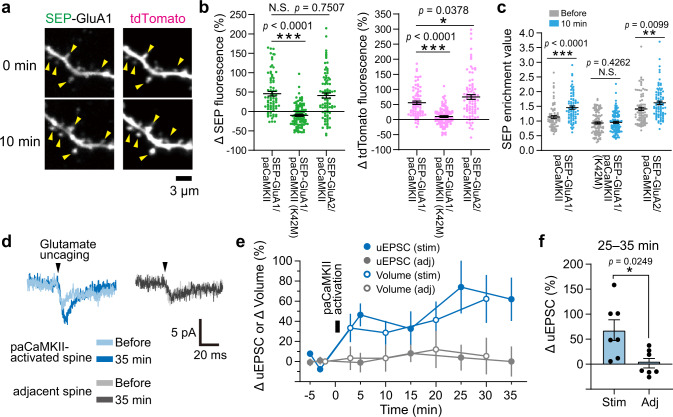
paCaMKII activation triggers AMPA receptor recruitment and functional LTP. **a** Two-photon fluorescence images of the dendritic spines of hippocampal neurons expressing SEP-GluA1 (left) and tdTomato-P2A-paCaMKII (right) before (top) and after (bottom) paCaMKII activation. Global laser scanning (15 × 15 µm) at 920 nm was used for both SEP/tdTomato imaging and paCaMKII activation. Note that paCaMKII was activated in whole view field. Yellow allow heads indicate spines. **b** Quantitative analysis of SEP fluorescence in spines (SEP-GluA1/2 recruitment into spine surface, left) and tdTomato fluorescence increase (spine volume increase, right) at 10 min after paCaMKII activation. **c** Spine enrichment values of SEP-GluA1 and -GluA2 before and after paCaMKII activation. *n*(spines/neurons) = 74/8 GluA1/paCaMKII, 101/8 GluA1/paCaMKII_K42M_, and 85/7 GluA2/paCaMKII. **d** Representative traces of uEPSC before and after paCaMKII activation in a targeted spine and their adjacent spine (averaged over five trails measured with 5 s intervals) in a neuron expressing tdTomato-P2A-paCaMKII. Note that single pulse of glutamate uncaging was used to evoke uEPSC, and the uEPSC before and after paCaMKII activation was compared in the same spine. **e** Averaged time courses of spine volume changes and the amplitude of uEPSC at −65 mV in the stimulated spines (stim) by paCaMKII activation and their adjacent spines (adj). *n*(spines/neurons) = 7/7 for both stim and adj. **f** Quantification of the amplitude of uEPSC in stimulated and adjacent spines averaged over 25–35 min following paCaMKII activation. The same data set with **e** was used. *n*(spines/neurons) is the same as in **e**. For **b**, **c**, **e**, and **f**, the data are presented as mean ± SEM. Statistical comparisons were performed using one-way ANOVA followed by Dunnett’s post-hoc test (**b**) and two-tailed paired *t* test (**c** and **f**). ***p < 0.001; **p < 0.01; *p < 0.05; N.S. not significant. Source data are provided as a Source Data file.

To investigate whether paCaMKII activation triggers recruitment of AMPA receptors into dendritic spines, we monitored surface GluA1 and GluA2 subunit expression using pH-sensitive super-ecliptic pHluorin (SEP)^46^. We cotransfected pyramidal neurons with tdTomato-P2A-paCaMKII and SEP-GluA1 or -GluA2. Then, we monitored tdTomato fluorescence inside the cells to measure the dendritic spine volume change and pH-sensitive SEP fluorescence on the cell surface as a measure of AMPA accumulation, as described previously^47^. By comparing the SEP fluorescence in spines before and 10 min after paCaMKII activation, we found that both SEP-GluA1 and -GluA2 fluorescence increased after paCaMKII activation with a concomitant increase in spine volume (Fig. 5a, b). In contrast, a kinase-dead paCaMKII_K42M_ mutant failed to increase SEP-GluA1 fluorescence in spines (Fig. 5a, b), suggesting that SEP-GluA1 recruitment is due to light-activated CaMKII.

Next, we calculated the spine surface density (enrichment value) change of SEP-GluA1 and -GluA2 (Fig. 5c, for calculation, see “Methods”) and found that the relative SEP-GluA1 and -GluA2 density increased 10 min after paCaMKII activation. In contrast, kinase-dead paCaMKII_K42M_ did not increase SEP-GluA1 enrichment or spine volume after 10 min, again suggesting that SEP-GluA1/-GluA2 recruitment is due to increased light-dependent kinase activity (Fig. 5b, c). Taken together, optogenetic paCaMKII activation leads to AMPA receptor recruitment into spines.

Since paCaMKII activation recruited AMPAR subunits into spines, we tested whether this activation also induces functional LTP. To do so, we used whole-cell patch-clamp recording to measure glutamate uncaging-evoked excitatory postsynaptic currents (uEPSCs) in the presence of Mg^2+^, which blocks NMDA currents (i.e., we monitored postsynaptic AMPA currents), in single spines (Fig. 5d). We monitored uEPSCs after paCaMKII activation and found that the uEPSC amplitude and spine volume were increased and sustained over 30 min in paCaMKII-activated spines, but not in adjacent spines (Fig. 5e, f). These data suggest that paCaMKII activation induces spine-specific functional LTP, likely due to AMPAR recruitment to the stimulated spines.

### Development of Cdc42 and RhoA FRET sensors

**Fig. 6:**
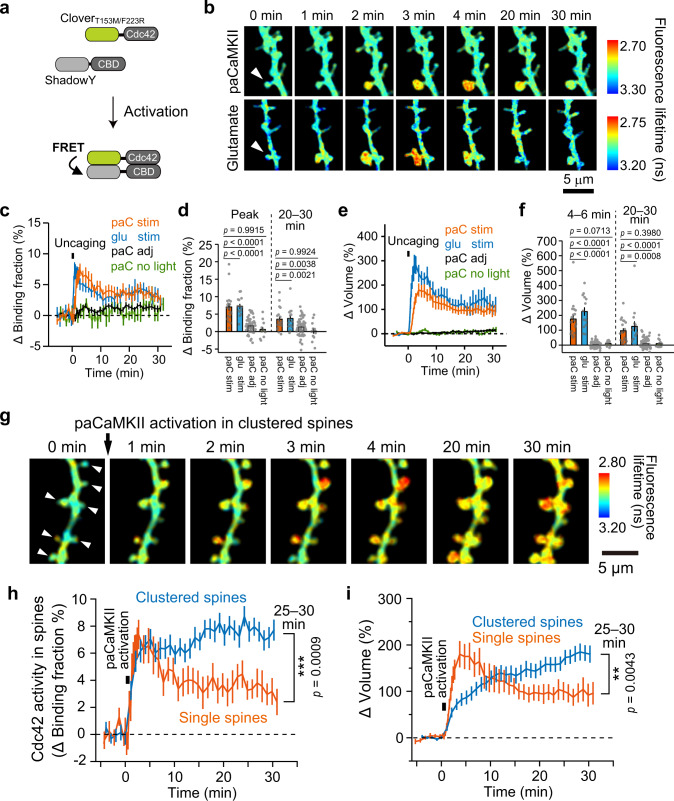
Spatiotemporal dynamics of Cdc42 activation upon single-/clustered-spine paCaMKII activation. **a** Schematic of Cdc42 FRET sensor activation. **b** Fluorescence lifetime images of Cdc42 during sLTP upon paCaMKII activation (top) and glutamate uncaging (bottom). Cdc42 FRET sensor and tdTomato-P2A-paCaMKII were coexpressed in hippocampal CA1 neurons in a cultured slice. Two-photon excitation at 1010 nm was used to excite Clover_T154M/F223R_, and 720/820 nm were used for glutamate uncaging and paCaMKII activation, respectively. The white arrows indicate stimulated spines. The warmer color indicates high Cdc42 activity. Averaged time courses of the binding-fraction change of Clover_T154M/F223R_-Cdc42 (Cdc42 activation) (**c**) and spine volume change (**e**) in glutamate-uncaged spines (glu stim) or paCaMKII-activated spines (paC stim), adjacent spines (paC adj; >2 µm of space between the stimulated spines), and nonstimulated spines (paC no light). *n*(spines/neurons) = 19/11 paC stim, 62/11 paC adj, 17/5 paC no light, 14/9 glu stim. **d**, **f** Quantification of transient (five-point rolling average at peak) (**d**, left) and sustained (averaged over 20–30 min) binding-fraction change (**d**, right). And quantification of spine volume change averaged over 4–6 min (**f**, left) and averaged over 20–30 min (**f**, right). The same data set with **c** and **e** was used for quantification. *n*(spines/neurons) is the same as in **c** and **e**, respectively. **g** Fluorescence lifetime images of Cdc42 activity upon paCaMKII activation in clustered (multiple) spines. paCaMKII was sequentially activated in five to eight spines for each neuron by two-photon excitation at 820 nm in indicated spines (white arrows). Averaged time courses of the binding fraction of Clover_T154M/F223R_-Cdc42 in spines (**h**) and spine volume change (**i**) following clustered-spine stimulation. *n*(spines/neurons) = 60/8. The time courses of single-spine stimulation in **c** and **e** (orange) was superimposed for comparison (orange). For all figures, the data are presented as mean ± SEM. Statistical comparisons were performed using one-way ANOVA followed by Dunnett’s post-hoc test (**d** and **f**) and two-tailed unpaired *t* test (**h** and **i**). ***p < 0.001; **p < 0.01; *p < 0.05; N.S. not significant. Source data are provided as a Source Data file.

The spatiotemporal activity pattern of the downstream molecules of CaMKII remains elusive^7^. To demonstrate that paCaMKII can be used to monitor the direct impact of CaMKII activation on downstream signaling, we monitored the activation of the small GTPases, Cdc42, and RhoA. We chose these molecules because while glutamate activates Cdc42/RhoA in a CaMKII-dependent manner^48,49^, it has not been known whether CaMKII activation is sufficient to activate these molecules. To monitor Cdc42 or RhoA activation upon paCaMKII activation, we developed new FRET sensors similar to those reported previously^48,50^. For the FRET donor, we fused the Cdc42 N-terminus to the yellow-green fluorescent protein Clover_T154M/F223R_ to preserve the ability of the Cdc42 C-terminus to associate with membranes. We used this Clover mutant because it can be excited and imaged by two-photon excitation at 1010 nm^48,50^, which does not activate paCaMKII. For the FRET acceptor, we fused the dark yellow fluorescent protein ShadowY to the N-terminus of the Cdc42-binding domain (CBD) (Pak3, residues 60–113 with S74A and F84A mutations)^48^. Whereas the CBD binds to activated Cdc42 (Kd ~1.8 µM), it does not bind to inactive Cdc42 (Kd ~149 µM)^48^. Since the CBD binds to activated Cdc42, Clover_T154M/F223R_-Cdc42 activation by GTP binding leads to its association with ShadowY-CBD, resulting in FRET between Clover_T154M/F223R_ and ShadowY (Fig. 6a). Thus, Cdc42 activation can be measured as a decrease in the fluorescence lifetime of Clover_T154M/F223R_. For the RhoA FRET sensor, we used a Clover_T154M/F223R_-RhoA fusion protein as a FRET donor and ShadowY fused to the RhoA-binding domain (RBD) (Rhotekin, residues 8–81) as a FRET acceptor (Supplementary Fig. 5a). While RBD binds to activated RhoA (Kd ~3.9 µM), it does not bind to inactive RhoA (Kd ~47 µM)^48^. These probes were coexpressed with tdTomato-P2A-paCaMKII in CA1 pyramidal neurons in cultured hippocampal slices and imaged by two-photon FLIM^37,48^.

### paCaMKII activation leads to the activation of Cdc42 rather than RhoA

We first verified that glutamate uncaging activate the Cdc42 and RhoA FRET sensors in dendritic spines as described previously (Fig. 6b–d, Supplementary Fig. 5b–d)^48,49^. After confirming that the FRET sensors worked, we activated paCaMKII in the spines of the same neurons with a low-frequency train of 820 nm two-photon excitation pulses (820 nm, 30 pulses, 0.5 Hz, 40 ms duration/pulse, 4 mW), which does not uncage caged glutamate. Both paCaMKII photoactivation and glutamate uncaging-induced sLTP, but the change in the initial phase was significantly smaller than sLTP induced by glutamate uncaging (4–6 min), while the late phase (20–30 min) was similar (Fig. 6e, f; Supplementary Fig. 5e, f). This difference suggests that CaMKII-independent signaling pathways exist in the initial phase of sLTP. Upon paCaMKII activation, Cdc42 activation occurred specifically in spines within a few minutes and was followed by elevated activity lasting more than 30 min, similar to that observed with glutamate uncaging (Fig. 6b–d). In contrast, paCaMKII activation hardly activated RhoA (Supplementary Fig. 5b–d). These findings suggest that CaMKII activation is sufficient for triggering Cdc42 activation, but not RhoA activation.

To test whether the fluorescence lifetime change of the Cdc42 and RhoA sensors were due to specific binding to their respective FRET acceptors, we performed control experiments in which the ShadowY-CBD and ShadowY-RBD acceptors were replaced with the ShadowY-CBD_H83L/H86L_ mutant, which does not bind Cdc42 or RhoA^51^. These “false acceptors” resulted in much smaller FRET changes during sLTP than the right sensors in response to optogenetic paCaMKII activation and glutamate uncaging (Supplementary Fig. 6a–h), suggesting that the FRET signal we observed with the true FRET acceptors was indeed due to donor/acceptor binding.

### paCaMKII activation in clustered spines leads to the enhanced Cdc42 activation and sLTP

Several lines of experiments have shown the occurrence of spatial clusters of synaptic plasticity^28^, and locally (~10 µm) synchronized NMDA receptor-dependent Ca^2+^ influx into dendritic spines in pyramidal neurons^52–54^. Moreover, NMDA-dependent Ca^2+^ influx induces CaMKII^5^ and Cdc42 activation^48^. These findings motivated us to monitor Cdc42 activation and sLTP upon CaMKII activation in clusters of spines on a short stretch (~15 µm) of dendrites.

We activated paCaMKII in clustered spines by two-photon excitation at 820 nm and imaged Cdc42 activity and spine volume by two-photon excitation at 1010 nm (Fig. 6g). paCaMKII activation in single and clustered spines induced a rapid increase in Cdc42 activity (Fig. 6h). But, in the late phase (25–30 min), paCaMKII activation in clustered spines induced enhanced Cdc42 activation, compared to single-spine stimulation (Fig. 6h). While paCaMKII activation in single spines induced a rapid volume change during sLTP, activation in clustered spines induced a more gradual increase over 30 min (Fig. 6i). In the late sLTP phase, the spine volume change by the clustered stimulation was significantly larger than that by single-spine stimulation (Fig. 6i). The slower volume increase in clustered spines may be due to the lack of sufficient resources, such as actin and their regulators, required for spine volume increase.

**Fig. 7:**
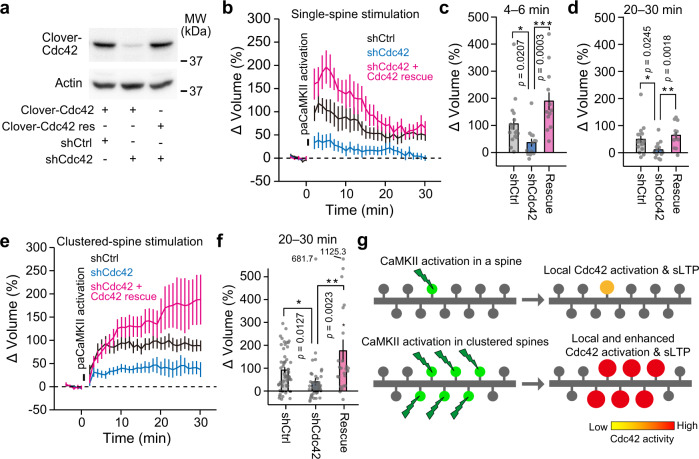
The Cdc42 downregulation suppresses sLTP induced by single- and clustered-spine stimulation. **a** Verification of shRNA. HeLa cells were transfected with control shRNA (shCtrl) or shRNA against Cdc42 (shCdc42), coexpressed with wild-type Clover-Cdc42 or its shRNA-resistant mutant (Clover-Cdc42res), respectively. The transfection of shCdc42 suppresses the expression of Clover-Cdc42 (lane 2 in the top panel). The transfection of shCdc42 does not suppress the expression of Clover-Cdc42res (lane 3 in the top panel). Averaged time courses of spine volume change upon single-spine stimulation (**b**) or clustered-spine stimulation (**e**) in neurons under manipulations of Cdc42 signaling. For single-spine stimulation, *n*(spines/neurons) = 17/6 shCtrl, 13/7 shCdc42, and 12/4 rescue. For clustered-spine stimulation, *n*(spines/neurons) = 51/6 shCtrl, 45/7 shCdc42, and 25/4 rescue. Quantification of transient (**c**, averaged over 4–6 min) and sustained (**d**, averaged over 20–30 min) spine volume change after single-spine stimulation. The data set used in **b** was analyzed. *n*(spines/neurons) is the same as in **b**. **f** Quantification of spine volume change (averaged over 20–30 min) after clustered-spine stimulation. The data set used in **e** was analyzed. *n*(spines/neurons) is the same as in **e**. **g** A model of CaMKII-dependent Cdc42 activation and its relationship with sLTP. Simultaneous activation of CaMKII in clustered spines leads to enhanced Cdc42 activity and sLTP. For all figures, the data are presented as mean ± SEM. Statistical comparisons were performed using two-tailed unpaired *t* test (**c**, **d**, and **f**). ***p < 0.001; **p < 0.01; *p < 0.05; N.S. not significant. Source data are provided as a Source Data file.

We confirmed the importance of CaMKII-dependent Cdc42 activation for sLTP by downregulating Cdc42 using short-hairpin (sh)RNA (Fig. 7a). Cdc42 downregulation suppressed sLTP induced by single- and clustered-spine stimulation (Fig. 7b–f). The phenotypes caused by the shRNA were rescued by coexpressing shRNA-resistant Cdc42, suggesting that the effect of the shRNA is specific (Fig. 7b–f). Thus, Cdc42 is required for paCaMKII-dependent sLTP in single and clustered spines (Fig. 7g).

### sLTP induction by paCaMKII activation in vivo

**Fig. 8:**
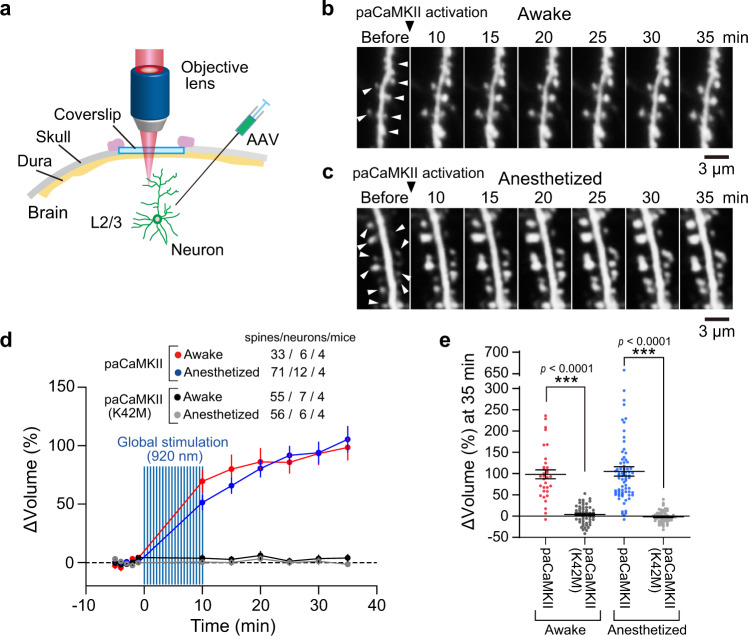
paCaMKII activation triggers sLTP in cortical neurons in vivo. **a** Schematic drawing of in vivo imaging. Using a DIO system in combination with a low expression of Cre, Clover was sparsely expressed in neurons and was excited by two-photon excitation at 1000 nm. **b**, **c** Two-photon images of dendritic spines of layer 2/3 pyramidal neurons of the sensory cortex. The paCaMKII was activated by global scanning with two-photon excitation at 920 nm. The images were taken in awake (**b**) and anesthetized (**c**) states. White arrows indicate the enlarged spines by paCaMKII activation. **d** Averaged time courses of spine volume change induced by paCaMKII activation. Data using the kinase-dead paCaMKII_K42M_ are also shown. The number of samples (spines/neurons/mice) is indicated in the figure. The data are presented as mean ± SEM. **e** Quantification of spine volume change at 35 min after paCaMKII activation. The same data set with (**d**) was used. *n*(spines/neurons) is the same as in **d**. The data are presented as mean ± SEM. ***p < 0.001; two-tailed unpaired *t* test. Source data are provided as a Source Data file.

An important advantage of optogenetic tools is that they can be applied noninvasively to living animals, such as mice. To confirm that paCaMKII activation can trigger sLTP in vivo, we expressed paCaMKII and Clover in layer 2/3 neurons of the mouse cortex by injecting adeno-associated viral vectors (AAVs) encoding paCaMKII and Clover (Fig. 8a). To visualize individual spines, we sparsely expressed Clover by using a double-floxed inverted open (DIO) reading frame system combined with low Cre expression. We imaged the neurons expressing Clover with a two-photon microscope and activated paCaMKII to induce sLTP at multiple spines by raster scanning in a view field with two-photon excitation (Fig. 8b, c). paCaMKII activation pulses induced sLTP over 30 min at multiple spines in awake and anesthetized mice (Fig. 8d, e), suggesting that paCaMKII can be used to induce sLTP in vivo.

## Discussion

The necessity of CaMKII for LTP has been extensively studied using various tools such as drugs, peptides, and siRNAs. However, it has been impossible to explore the sufficiency of CaMKII activation in a single dendritic spine. We describe a new genetically encoded single-molecule-type paCaMKII, which can be activated in single spines using two-photon excitation. paCaMKII activation is sufficient to induce synaptic plasticity at the single-synapse level both in vitro and in vivo. Although a previous study found 140 binding protein partners of Ca^2+^-calmodulin^55^, one of its partners, CaMKII, is sufficient for LTP. Our findings indicate that paCaMKII is a uniquely useful tool to dissect the effect of CaMKII activation on the downstream signaling required for LTP in single dendritic spines. Further, paCaMKII can be used to elucidate other processes that depend on this kinase.

Light illumination induces paCaMKII structural changes and autophosphorylation, suggesting that photoactivation of paCaMKII can substitute for Ca^2+^/CaM binding to the subunit acting as kinase and substrate (i.e., T286 phosphorylation). This suggests that paCaMKII works similarly to endogenous CaMKII. Indeed, paCaMKII can also be integrated into an oligomer with endogenous CaMKII. One concern is the effect of CaMKII overexpression. For example, since paCaMKII is activated by NMDA in a Ca^2+^-dependent manner, Ca^2+^-dependent paCaMKII activity may disturb resting CaMKII signaling. However, this effect should be limited because the paCaMKII expression level in our experiments relatively low (~15 μM) compared to endogenous CaMKII (~60 μM in proximal dendrites)^56^. Since the holoenzyme is expected to consist of 4:1 endogenous CaMKII to paCaMKII, it may not have a profound effect on the signaling.

The simultaneous activation of adjacent kinases in an oligomer is essential for autophosphorylation. Since paCaMKII forms an oligomer with endogenous CaMKII subunits and light illumination activates paCaMKII but not endogenous subunits, the chance that the adjacent subunits will be activated at the same time by autophosphorylation is low, which may reduce the efficiency of autophosphorylation. However, single-spine paCaMKII activation induced spine enlargement comparable to that induced by glutamate uncaging, implying that sufficient autophosphorylation occurs. We speculate phosphorylation between the inter-oligomer or between the remote subunits within the oligomer may occur in addition to autophosphorylation between adjacent subunits. It may be facilitated by the insertion of LOV2, which extends the effective radius of kinase activity^10,34^.

As a tool to induce sLTP at the single-spine level, paCaMKII has several superior characteristics to widely used MNI-glutamate uncaging. First, since MNI-glutamate can have antagonistic effects^57^ and requires bath or local puff application at high concentrations (~ mM), it may have side effects on surrounding cells. In contrast, paCaMKII can be expressed at the single-cell level and should have no side effects on neighboring cells. Second, while glutamate uncaging experiments require the removal of extracellular Mg^2+^ or depolarization of neurons to release Mg^2+^ from NMDARs^2^, paCaMKII can be activated in physiological conditions such as the mouse brain in vivo. Third, the wavelength window of MNI-glutamate uncaging is up to 800 nm^2^, while that of paCaMKII is up to ~980 nm (i.e., FMN absorption)^32^. Because a longer wavelength has a superior penetration depth, this is advantageous for sLTP induction in deep tissues. Fourth, since the two-photon cross section of LOV2 (0.5–0.9 GM in 800–900 nm)^32^ is higher than that of MNI-glutamate (0.06 GM at 720 nm, the most widely used wavelength for efficient photolysis)^58,59^, weaker laser power (i.e., lower cell toxicity) can be used for sLTP induction. Together, these advantages facilitate the manipulation of synaptic plasticity under physiological conditions with low phototoxicity.

Although paCaMKII activation and glutamate uncaging result in similar outcomes, i.e., spine volume change, AMPAR recruitment, and enhanced EPSCs, there are some differences. One important difference is the dependency of sLTP on protein synthesis. Previous reports found that sLTP induced by glutamate uncaging is independent of protein synthesis, but is dependent on pairing with postsynaptic spikes^40^ or forskolin^41^. We found that sLTP induced by paCaMKII activation depends on protein synthesis without postsynaptic spikes or forskolin.

We also found that the time course of spine volume change induced by paCaMKII activation and glutamate uncaging were slightly different. The spine volume change in the early phase (4–6 min) induced by paCaMKII activation was smaller than the volume change induced by glutamate uncaging. This difference might be explained by the fact that paCaMKII activates a single pathway, while glutamate activates multiple signaling pathways.

Using paCaMKII, we found that paCaMKII activation leads to Cdc42 activation. However, the signal transduction pathway linking CaMKII to Cdc42 remains unknown^7^. Cdc42 is activated by guanine nucleotide exchange factors (GEFs) and is inactivated by GTPase-activating proteins (GAPs). One possible mechanism is that CaMKII phosphorylates these GEFs, and thus increases their ability to activate Cdc42. Second, CaMKII activation recruits GEFs into dendritic spines and activates Cdc42, similar to the Rac GEF Tiam1 and RhoA GEF Lfc, which translocate into spines upon neuronal stimulation^15,60^. Another possibility is that CaMKII activation causes Cdc42 GAP removal from spines, as seen for SynGAP, which is phosphorylated by CaMKII during LTP, resulting in rapid dispersal of SynGAP from spines^61^. Similar dispersal of Cdc42 GAPs from spines in response to CaMKII activation might shift the local GEF/GAP equilibrium, leading to Cdc42 activation.

We report that paCaMKII activation in clustered spines enhances Cdc42 activation and sLTP when compared to activation in single spines. One mechanism to explain this may be synaptic crosstalk due to intracellular signal spreading to neighboring spines. Previous studies found that Ras, RhoA, and Rac1 activation is CaMKII dependent^15,48,49,62^, and the spreading of activation to adjacent spines lowers the threshold for sLTP induction^49,62^. If such events occur cooperatively in clustered spines, they may lead to enhanced or sustained Cdc42.

In summary, we have developed a paCaMKII that allows selective CaMKII pathway activation. One potential future direction is to combine paCaMKII with FRET-based signaling sensors. Here, we demonstrated that paCaMKII can be combined with FRET imaging by 2pFLIM-FRET. Since many types of FRET sensors are available, we should be able to directly identify the various downstream signaling molecules of CaMKII and their spatiotemporal profiles. Also, since we can selectively activate the CaMKII pathway, it may be possible to perform CaMKII activity-dependent gene/protein expression profiling by combining transcriptomics/proteomics approaches.

Recently, a Rac1-based photoactivatable optoprobe enabled the induction of shrinkage of the subsets of activated spines in vivo^21^. Since paCaMKII allows LTP induction at the level of single synapses, we should be able to combine paCaMKII with activated-synapse tagging technology for manipulating specific subsets of spines in vivo. Such technology will allow the identification of the direct link between synaptic plasticity and animal behavior.

## Methods

### Animals

All animal procedures were approved by the National Institutes of Natural Sciences Animal Care and Use Committee and were performed under the relevant guidelines and regulations. All dissociated cultures were prepared from C57BL/6N mice (SLC). All slice cultures were prepared from Wistar rats (SLC or Charles River). This study used dissociated and slice cultures from both male and female pups. For in vivo imaging, the experiments were performed using male and female adult (2 months) C57BL/6N mice (Charles River).

### Reagents

Anisomycin and N-methyl-D-aspartic acid (NMDA) were purchased from Sigma-Aldrich (St. Louis, MO, USA). AAV1-CaMKII-Cre was purchased from the University of Pennsylvania (U Penn; Philadelphia, PA, USA). Cycloheximide was from Abcam (Cambridge, UK). MNI-caged L-glutamate (MNI-caged glutamate) was from Tocris Bioscience (Bristol, UK).

### Plasmids

Plasmids containing *CaMKIIα*, *Cdc42/RhoA/Pak*, *SEP-GluA1/2*, *Rhotekin*, *WPRE*, and *CaMKII0.4* promoter genes are gifts from Y. Hayashi, M. Matsuda, S. Soderling, G. Bokock, K. Kobayashi, and M. Ehlers, respectively. *GCaMP6f*, *Clover*, *LOV2(SD)*, *CAMKIIβ*, *Cre*, *WPRE3* genes, and pAAV-hSyn-DIO-EGFP plasmid were gifts from D. Kim, M. Lin, K. Hahn, T. Meyer, C. Cepko, B.K. Kaang, and B. Roth (Addgene plasmid #40755, #40255, #81033, #21227, #13775, #61463, #50457), respectively. The synthesized gene encoding codon-optimized *LOV2*_408–546_ gene was purchased from Integrated DNA Technologies (Coralville, IO, USA). pAAV-RC-DJ (AAV2/DJ) and pAAV-MCS/pAAV-Helper were purchased from Cell Biolabs (San Diego, CA, USA) and Agilent Technologies (Santa Clara, CA, USA), respectively.

paCaMKIIα was constructed by inserting the codon-optimized LOV2_408–546_ sequence between the kinase and association domains of rat CaMKIIα as described in the main text. The introduction of mutations was carried out by using the QuikChange Site-Directed Mutagenesis Kit (Agilent Technologies). The CMV-tdTomato-P2A-paCaMKIIα plasmid was constructed by inserting tdTomato^63^ and paCaMKIIα, together with P2A^64^ sequences ATNFSLLKQAGDVEENPGP into the modified pEGFP-C1 vector by replacing EGFP.

The CMV-mEGFP-paCaMKIIα and CMV-Flag-paCaMKIIα plasmids were constructed by inserting mEGFP (EGFP with A206K monomeric mutation)^65^ or Flag with paCaMKIIα into the modified pEGFP-C1 vector by replacing EGFP. The CMV-mEGFP-paCaMKIIα-ShadowG was constructed by inserting mEGFP, paCaMKIIα, and ShadowG^66^ into the modified pEGFP-C1 vector by replacing with EGFP.

For construction of the Cdc42 FRET sensor (CMV-ShadowY-CBD-P2A-Clover_T154M/F223R_-Cdc42), we fused ShadowY (a.a. residues 1–232)^50^ as an acceptor fluorescent protein to the N-terminus of the activated CBD of Pak3 (a.a. residues 60–113 with two mutations: S74A and F84A)^48^ via the linker peptide RSRG. Subsequently, Cdc42 fused to Clover_T154M/F223R_ as a donor fluorescent protein via the linker peptide SGLRSRG was fused to the C-terminus of the acceptor protein via the P2A sequence so that the CBD and Cdc42 parts were translated into different polypeptides within the cell.

The RhoA FRET sensor (CMV-ShadowY-RBD-P2A-Clover_T154M/F223R_-RhoA) was constructed similarly to the Cdc42 FRET sensor using an activated RhoA-binding domain (RBD) of rhotekin (a.a. residues 8–89).

The following plasmids pAAV-CaMP0.4-DIO-Clover-WPRE3 and pAAV-CaMP0.4-Flag-His×6-paCaMKIIα were constructed by inserting the respective components in the pAAV-MCS.

shRNAs were prepared using the custom pSuper vector (Oligoengine). The following target sequences were used for shRNA (5′-3′): CAGTCACAGTTATGATTGGT for Cdc42 (rat, mouse, and human), and ATCGATCGAAAGATCGCTC for control. shRNA-resistant Clover-Cdc42 was prepared by introducing six silent mutations in the targeted sequences.

### AAV production and purification

Serotype DJ AAVs were produced and purified as described previously^67^. Briefly, HEK293 cell culture was maintained on 15-cm plates in Dulbecco’s modified Eagle’s medium (DMEM) supplemented with 5% fetal bovine serum (FBS) with no antibiotics at 37 °C and 5% CO_2_. Six 15-cm dishes at 70% confluency were prepared for polyethylenimine (PEI) transfection. For PEI-based transfection, plasmids at a ratio of 1:1.6:1 (45 µg of a transgene in pAAV, 72 µg of pAAV-Helper, and 45 µg of pAAV-RC-DJ [AAV2/DJ]) were used for six dishes. The PEI–DNA complex at the ratio of 4:1 (w/w) (PEI “MAX” [648 µg, Cosmo Bio; Tokyo, Japan] and DNA [162 µg]) was incubated in 6 ml of serum-free DMEM for 10 min at RT, making DMEM–PEI–DNA mixture. Subsequently, the mixture was diluted with 150 ml of serum-free DMEM. The culture medium in the dishes was decanted and replaced with the DMEM–PEI–DNA mix (25 ml per dish), subsequently, the dishes were incubated at 37 °C and 5% CO_2_ for 96 h. Before the collection of supernatants, NaCl was added to 500 mM to increase the solubility of AAV particles and further incubated 30 min at 35 °C and 5% CO_2_.

The collected culture medium (~150 ml) was centrifuged at 4000 × *g* for 20 min to remove cell debris, and the supernatant was filtered through a 0.22 µm pore. The clarified supernatant containing AAV was concentrated by the cross-flow cassette (Vivaflow 50, 100,000 MWCO, Sartorius; Göttingen, Germany) to about 15 ml. The AAV solution was further concentrated by Amicon Ultra-15 (100,000 MWCO, Merck; Kenilworth, NJ, USA) to about 4 ml, and incubated with benzonase at 250 U/ml at 37 °C for 1 h. Iodixanol step gradients were performed as described by Addgene (homepage section: AAV Purification by Iodixanol Gradient Ultracentrifugation). The buffer solution of the virus was exchanged with phosphate-buffered saline (PBS) during the concentration process.

The titer of AAVs was determined by quantitative PCR (qPCR) using THUNDERBIRD qPCR Mix (Toyobo; Osaka, Japan) and LightCycler 96 (Roche; Basel, Switzerland) according to the manufacturers’ protocols. The primers used for qPCR were as follows: 5′-acctctggattacaaaatttgtgaaag-3′ and 5′-aaccaggatttatacaaggaggagaaaatg-3′, which anneal to both WPRE3 and WPRE. The resultant virus titers typically ranged between 2 × 10^9^ and 2 × 10^10^ genome copies/µl in a total volume of 400 μl.

### Purification of tdTomato and the estimation of tdTomato concentration in neurons

His-tagged tdTomato was inserted into pRSET bacterial expression vector (Invitrogen; Carlsbad, CA, USA). Protein was overexpressed in *Escherichia coli* (DH5α) and purified with a Ni^+^-nitrilotriacetate column (HiTrap, GE Healthcare; Chicago, IL, USA), and desalted with a desalting column (PD10, GE Healthcare) equilibrated with PBS. The concentration of the purified protein was measured by the absorbance of the fluorophore (tdTomato, A_554_ = 138,000 cm^−1^ M^−1^)^63^. The concentration of tdTomato in neurons was estimated by measuring the fluorescence intensity of tdTomato in thick apical dendrites (3–6 µm in diameter) relative to that of purified tdTomato (10 µM) under a two-photon microscope.

### HeLa cell culture and transfection

HeLa cells were cultured in DMEM supplemented with 5% FBS at 37 °C in 5% CO_2_. The cells in 3-cm dishes were transfected with the plasmids using Lipofectamine 3000 (Invitrogen), followed by incubation for 16–22 h in the absence of serum. 2pFLIM-FRET imaging was conducted in HEPES-buffered artificial cerebrospinal fluid (HACSF; 30 mM HEPES, 130 mM NaCl, 2.5 mM KCl, 1 mM CaCl_2_, 1 mM MgCl_2_, 1.25 mM NaH_2_PO_4_, 25 mM glucose, pH 7.3) at 24–26 °C.

### Primary neuronal culture and AAV infection

Low-density cultures of dissociated embryonic cortical and hippocampal neurons were prepared as described previously^23^. Briefly, hippocampi or cortices were removed from C57BL/6N mice at embryonic days 18 and treated with papain for 10 min at 37 °C, followed by gentle trituration. Mouse cortical or hippocampal neurons were seeded onto PEI-coated 3-cm dishes (2 × 10^5^ cells/dish) and cultured in neurobasal medium (Gibco, Thermo Fisher; Waltham, MA, USA) supplemented with B-27 and 2 mM glutamax (Gibco). At DIV 9–11, primary neuronal cultures were infected with AAV-DJ particles at the concentration of 2.5 × 106 genome copies/ml. After ~72 h, the biochemical assay was carried out.

### Biochemical assay of autophosphorylation and oligomerization

For the paCaMKII autophosphorylation assay in HeLa cells, the culture medium was replaced with HACSF and incubated for 20 min at RT before the experiment. For cultured dissociated neurons, 1 µM of tetrodotoxin (TTX) was added to the culture medium and incubated for 30 min in the CO_2_ incubator. Subsequently, the culture medium was replaced with HACSF containing 1 µM of TTX and incubated for 20 min at RT before the experiment. To induce autophosphorylation, the samples in 3-cm dishes were continuously illuminated with a light-emitting diode (LED) (M455L2-C1, Thorlabs; Newton, NJ, USA) at 3 mW cm^−2^ for 2–5 min. The reactions were stopped at the indicated time by adding a lysis solution (50 mM Tris pH 7.5, 1% NP-40, 5% glycerol, 150 mM NaCl, 4 mM EDTA, 1 tablet/10 ml PhosSTOP (Sigma-Aldrich)). The samples were collected and centrifuged, the supernatant was dissolved in SDS sample buffer and subsequently analyzed by western blotting.

For NMDA-induced autophosphorylation assays, neurons were first washed with Mg^2+^-free artificial cerebrospinal fluid (ACSF; 127 mM NaCl, 2.5 mM KCl, 4 mM CaCl_2_, 25 mM NaHCO_3_, 1.25 mM NaH_2_PO_4_, and 25 mM glucose) containing 300 µM glycine, and 1 mM EDTA to remove Mg^2+^ bound to NMDA receptors. Subsequently, ACSF containing 20 µM NMDA and 300 µM glycine was applied and incubated for 2 min at RT. The cells were lysed with the lysis solution and the samples for western blotting were prepared as described above.

For the pull-down assay, dissociated cortical neurons expressing Flag-His×6-paCaMKII were lysed in lysis buffer (1% Triton X-100, 50 mM Tris-HCl, pH 7.5, 150 mM NaCl, 4 mM EDTA, 5% glycerol) and centrifuged. The total lysate was pooled from the supernatants, and the remaining supernatant was incubated with Ni sepharose (GE Healthcare) for 1 h at 4 °C. Samples were washed three times with wash buffer (20 mM Tris-HCl, 150 mM NaCl, 2 mM MgCl_2_, 50 mM imidazole, pH 7.5) and proteins were released with wash buffer containing 500 mM imidazole and dissolved in SDS sample buffer.

Western blotting was performed with the following antibodies: anti-Phospho-CaMKII (Thr286) (D21E4, Cell Signaling Technology); anti-CaMKIIα (6G9; Cell Signaling Technology); anti-CaMKIIβ (ab34703; Abcam); anti-GFP (M048-3; MBL); anti-Cdc42 (11A11; Cell Signaling Technology); anti-β-Actin (8H10D10; Cell Signaling Technology); anti-RFP (1G9; MBL) and HRP-anti-mouse and -rabbit (Jackson Laboratory; Bar Harbor, ME, USA). Uncropped western blot images are presented in Supplementary Fig. 7.

### Organotypic hippocampal and cortical slices and gene gun transfection

Hippocampal and cortical slices were prepared from postnatal day 5–9 Wistar rats as described^68^. Briefly, we deeply anesthetized the animal with isoflurane, after which the animal was quickly decapitated, and the brain removed. For hippocampal slices, the hippocampi were isolated and cut into 350 µm sections in ice-cold dissection medium (25 mM HEPES, 2 mM NaHCO_3_, 4 mM KCl, 5 mM MgCl_2_, 1 mM CaCl_2_, 10 mM D-glucose, 248 mM sucrose). For cortical slices, the cortex was isolated and cut in coronal sections. The slices were cultured on the membrane inserts (PICM0RG50, Millipore; Darmstadt, Germany) placed on culture medium (50% MEM, 21% HBSS, 15 mM NaHCO_3_, 6.25 mM HEPES, 10 mM D-glucose, 1 mM L-glutamine, 0.88 mM ascorbic acid, 1 mg/mL insulin, 25% horse serum), and incubated at 35 °C in 5% CO_2_.

After 7–12 days in slice culture, neurons were transfected with a gene gun (Scientz Biotechnology; Ningbo, China) using 1.6 µm gold particles coated with plasmids and imaged after 2–5 days. For making bullets, gold particles (2–4 mg) and DNA (4–16 µg) were used for a 30 cm long tube. For shRNA experiments, neurons were transfected with control shRNA (shCtrl, black), shRNA against Cdc42 (shCdc42, blue), and shRNA against Cdc42 with shRNA-resistant Clover-Cdc42 (Rescue, magenta). CA1 pyramidal neurons of cultured rat hippocampal slices (DIV 8–10) were transfected with biolistic gene transfer. For making bullets for a 30 cm long tube, DNA containing tdTomato-P2A-paCaMKII (20 µg) with shCtrl (10 µg) or shCdc42 (10 µg) plasmids was used. For rescue experiments, shRNA-resistant Clover-Cdc42 (5–7.5 µg) plasmid was further added. After 5 days of transfection, the experiments were carried out.

### Ca^2+^ imaging in spines

Ca^2+^ imaging in hippocampal slice cultures was performed using a custom two-photon microscope. A Ti:sapphire laser (Spectra-Physics; Santa Clara, CA, USA) tuned to 1000 nm was used for the excitation of both GCaMP6f and tdTomato. Fluorescence signals of GCaMP6f and tdTomato collected with a ×60, NA1.0 objective lens (Olympus; Tokyo, Japan) were divided with a dichroic mirror (565DCLP, Chroma; Taoyuan City, Taiwan). GCaMP6f fluorescence was detected by a photomultiplier tube (H7422-40p, Hamamatsu; Hamamatsu, Japan) through an emission filter (FF01-510/84; Chroma). The fluorescence of tdTomato to monitor spine volume change was detected by a photomultiplier tube (R3896; Hamamatsu) through an emission filter (FF01-625/90, Semrock; Rochester, NY, USA). The acquired signals were processed using a data acquisition board (PCI-6110, National Instruments; Austin, TX, USA) and ScanImage software^69^. For image acquisition, 128 × 32 pixels were acquired at 15.6 Hz. To induce sLTP at single spines, bath-applied 2 mM MNI-caged glutamate was uncaged by a second Ti:sapphire laser at a wavelength of 720 nm (30 trains, 0.5 Hz, 6 ms duration/pulse, 6–8 mW) near a spine of interest. paCaMKII was uncaged by a second Ti:sapphire laser at a wavelength of 900 nm (30 trains, 1 Hz, 80 ms duration/pulse, 4 mW) in a spine of interest. Laser power was measured under the objective lens. Two-photon glutamate uncaging was carried out in ACSF containing no MgCl_2_, 4 mM CaCl_2_, 1 µM TTX, and 2 mM MNI-caged L-glutamate aerated with 95% O_2_/5% CO_2_ at 24–26 °C.

### Two-photon paCaMKII uncaging

To activate paCaMKII in single or clustered spines with two-photon excitation, a second Ti:sapphire laser tuned at a wavelength of 820 or 900 nm was used with 30 trains (0.5 Hz or 1 Hz, 40–80 ms duration/pulse, 4 mW) in a spine of interest. Since the focal plane of imaging (1000–1010 nm) and uncaging (820–900 nm) lasers were different (0.5–1.0 µm) due to chromatic aberration in the microscope, it was compensated by moving sample stage in *z*-axis (0.5–1.0 µm) with piezo stages (PKVL64F-100U, NCS6101C, Kohzu; Kawasaki, Japan) during the light activation of paCaMKII.

For global paCaMKII activation, photoactivation was done by raster scan (15 × 15 µm^2^ region; three planes with 2-μm z-step were stacked and each plane was scanned 24 times) at 900 or 920 nm (laser power 4 mW, scan speed 7.5 µm/ms). For the experiment, ACSF containing 2 mM MgCl_2_ and 2 mM CaCl_2_ was aerated with 95% O_2_/5% CO_2_ at 24–26 °C.

For MNI-caged glutamate uncaging, a second Ti:sapphire laser tuned at a wavelength of 720 nm was used in extracellular solution with a train of 6 ms and 6–8 mW pulses (30 trains at 0.5 Hz) near a spine of interest. Experiments were performed in ACSF containing no MgCl_2_, 4 mM CaCl_2_, 1 μM TTX, and 4 mM MNI-caged L-glutamate aerated with 95% O_2_/5% CO_2_. Experiments were performed at 24–26 °C.

### Two-photon fluorescence lifetime imaging

Details of two-photon FLIM-FRET imaging were described previously^37^. Briefly, mEGFP or Clover_T154M/F223R_ in the FRET sensor was excited with a Ti:sapphire laser (Mai Tai; Spectra-Physics). The scanning mirrors were controlled with the ScanImage software^69^. The green fluorescence photon signals were collected by an objective lens (×60, 1.0 NA; Olympus) and a photomultiplier tube (H7422-40p; Hamamatsu) placed after a dichroic mirror (565DCLP; Chroma) and emission filter (FF01-510/84; Semrock). Measurement of fluorescence lifetime was conducted using a time-correlated single-photon counting board (SPC-150, Becker & Hickl GmbH; Berlin, Germany) controlled with custom software^37^. For the construction of a fluorescence lifetime image, the mean fluorescence lifetime in each pixel was translated into a color-coded image^70^. Analysis of the lifetime change and binding-fraction change was conducted as described elsewhere^70^.

To measure the light-dependent structural change of paCaMKII expressed in HeLa cells, the culture medium was replaced with HACSF before the observation under 2pFLIM-FRET. HeLa cells expressing mEGFP-paCaMKII-ShadowG or its mutants were imaged with a Ti:sapphire laser tuned to 920 nm at the power of 1 mW. To activate paCaMKII, the samples were continuously illuminated by a blue LED (470 nm LED; CoolLED) with a bandpass filter (FF01-469/35-25; Chroma) at 35 mW cm^−2^ for 2 s.

To measure Rho GTPase (Cdc42/RhoA) activity in hippocampal slice culture, Cdc42 (CMV-ShadowY-CBD-P2A-Clover_T154M/F223R_-Cdc42) or RhoA FRET sensor plasmids (CMV-ShadowY-RBD-P2A-Clover_T154M/F223R_-RhoA) were cotransfected with CMV/CaMP0.4-tdTomato-P2A-paCaMKII plasmids by gene gun. To simultaneously activate Clover_T154M/F223R_ and tdTomato, a Ti:sapphire laser tuned to 1010 nm in a range of 0.7–1.7 mW was used.

### Analysis of the fluorescence lifetime image

To generate fluorescence lifetime images, we acquired the mean fluorescence lifetime in each pixel by calculating the mean photon arrival time <*t* > using the following equation:

1 $$ < t > = \smallint tF\left( t \right)dt \div \smallint F\left( t \right)dt - t_0$$

where *t*_*o*_ is obtained by fitting the whole image with single exponential or double exponential functions convolved with an instrument response function as described previously^70^. Subsequently, the mean fluorescence lifetime in each pixel was converted to the corresponding color. FRET efficiency and the binding fraction (fraction of the donor fluorescent protein undergoing FRET) were calculated as in other studies^37,70^.

### Calculation of the AMPA receptor insertion

The fluorescence of SEP and tdTomato was monitored and compared before and after induction of sLTP at multiple spines using raster scanning (15 × 15 µm). Imaging SEP and tdTomato fluorescence and the induction of the sLTP by paCaMKII were simultaneously carried out by two-photon laser scanning at 920 nm (3–11 mW). To assess the incorporation of SEP fused AMPA receptor subunits GluA1/GluA2 into spines, the density of spine surface subunits as an enrichment value was also measured (Fig. 5c)^47^. First, tdTomato fluorescence was converted to two-dimensional using the following equation by assuming that spine heads are spherical:

2 $$R_{\mathrm{spine}\_\mathrm{surface}} = (4\pi )^{\frac{1}{3}} \times ( {3R_{\mathrm{spine}\_\mathrm{volume}}} )^{\frac{2}{3}}$$

SEP fluorescence (*G*_spine_surface_) was divided by *R*_spine_surface_ to measure the relative density of GluA1/GluA2. To compare across different cells, the spine enrichment value was further divided by the dendritic enrichment value (i.e., *G*_dendrite_surface_/*R*_dendrite_surface_), where *R*_dendrite_surface_ is calculated by the above equation by replacing *R*_spine_surface_ with *R*_dendrite_surface_ and *R*_spine_volume_ with *R*_dendrite_volume_, respectively. Since the dendrites are not spherical structure, *R*_dendrite_surface_ is an approximate calculation.

### Electrophysiology

Whole-cell patch clamping was performed with patch pipettes (9–12 MΩ). We used cells with series resistance lower than 40 MΩ for experiments. CA1 pyramidal neurons of cultured rat hippocampal slices (DIV 8–10) were transfected with biolistic gene transfer using gold beads coated with plasmids containing cDNA of tdTomato-P2A-paCaMKII (16 µg). To induce LTP with paCaMKII uncaging (920 nm, 30 trains, 0.5 Hz, 80 ms, 4 mW), we performed experiments in voltage-clamp mode using Cs^+^ internal solution (130 mM CsMeSO_3_, 10 mM Na-phosphocreatine, 4 mM MgCl_2_, 4 mM Na_2_-ATP, 0.4 mM Na_2_-GTP, 10 mM HEPES, and 200 μM Alexa Fluor 488 hydrazide (Invitrogen), pH 7.3) under a two-photon microscope with a 40 × 0.8 NA objective lens (Olympus), and measured two-photon glutamate uEPSC at a spine through the patch pipette using a patch-clamp amplifier (MultiClamp 700B, Molecular Devices; San Jose, CA, USA). Experiments were performed in a buffer (136 mM NaCl, 5 mM KCl, 0.8 mM KH_2_PO_4_, 20 mM NaHCO_3_, 1.3 mM L-glutamine, 0.2 mM ascorbic acid, 2 mM CaCl_2_, 2 mM MgCl_2_, MEM amino acids solution (Gibco), MEM vitamin solution (Gibco), 1.5 mg/ml phenol red) containing 1 µM TTX and 2–3 mM MNI-caged glutamate aerated with 95% O_2_/5% CO_2_ at 24–26 °C.

### Imaging and paCaMKII uncaging in vivo

For viral infection, mice were anesthetized with an intraperitoneal injection of ketamine (70 mg/kg) and xylazine (10.5 mg/kg) and were secured on a stereotaxic frame (Narishige; Amityville, NY, USA). To inject the viruses into the primary somatosensory cortex (S1), the skull above the S1 (0.5 mm caudal from bregma, 1.5 mm lateral from the middle) was thinned (1 mm diameter) with a drill for the AAV-containing glass pipette insertion. A glass pipette was slowly inserted to a depth of 350 µm (layer 2/3) from the surface of the cortex. Approximately 250 nl of a viral solution was injected at a rate of 25 nl min^−1^. To sparsely label neurons with Clover, we used a DIO reading frame system in combination with a lower amount of Cre expression. The mixture of the following AAV vectors was infected with the genome titer ratio of 1:900:900, CaMKII-Cre (U Penn), CaMP0.4-Flag-His×6-paCaMKII, and CaMP0.4-DIO-Clover-WPRE3. After AAV injection, the incision was sealed with a surgical staple, and mice were returned to their home cage and housed until the imaging sessions started. We typically waited ~4 weeks after AAV injection until a sufficient level of Clover expression was obtained.

To construct a cranial window for imaging, we anesthetized mice with isoflurane (3% for induction, 1% for surgery), and a custom-made head plate was attached to the skull. A cranial window was made above the S1 region where AAV plasmids were injected, and the dura mater was removed. The exposed S1 region was covered with double cover glass (top: diameter 4.0 mm, bottom: 2 × 2 mm, Matsunami; Bellingham, WA, USA). The coverslips were secured with adhesive glue and dental cement.

For in vivo two-photon imaging, dendritic spines of cortical neurons in awake mice or mice anesthetized with 1% isoflurane were observed using a two-photon microscope (A1R MP, Nikon; Tokyo, Japan) with a water immersion objective lens (×25, 1.1 NA, Nikon). The imaging locations of dendritic spines were 100 μm below the cortical surface (layer 1). Images of dendritic spines of neurons expressing Clover were taken every 1 min (61 × 30 µm^2^ rectangle region, 5–12 planes with 0.5 μm z-step were stacked, each plane was scanned once) at the wavelength of 1000 nm (laser power 8–15 mW). For global two-photon paCaMKII activation, 5–12 planes (61 × 30 µm^2^ region, each plane was scanned once) with 0.5 µm apart to *z*-axis were scanned at 920 nm (laser power 12–29 mW) every 30 s for 10 min. Subsequently, the dendritic spines were observed at 1000 nm every 5 min for 25 min. The images were analyzed by ImageJ (National Institutes of Health; Bethesda, MD, USA).

### Quantification and statistical analysis

Statistical analysis was performed using the Matlab or GraphPad Prism software. The types of statistical tests, the number of samples, and statistical significance are described in the figures or legends.

### Reporting summary

Further information on research design is available in the Nature Research Reporting Summary linked to this article.

## Supplementary information

Supplementary Information

Peer Review File

Reporting summary

## Data Availability

Image data will be available upon reasonable request. Source Data are provided with this paper.

## Source data

Source Data

## Materials availability

Further information and requests for resources and reagents should be directed to and will be fulfilled by H.M. (murakosh@nips.ac.jp).

## References

[1] Nakahata Y, Yasuda R (2018). Plasticity of spine structure: local signaling, translation and cytoskeletal reorganization. Front. Synaptic Neurosci..

[2] Matsuzaki M, Honkura N, Ellis-Davies GC, Kasai H (2004). Structural basis of long-term potentiation in single dendritic spines. Nature.

[3] Malinow R, Malenka RC (2002). AMPA receptor trafficking and synaptic plasticity. Annu. Rev. Neurosci..

[4] Derkach VA, Oh MC, Guire ES, Soderling TR (2007). Regulatory mechanisms of AMPA receptors in synaptic plasticity. Nat. Rev. Neurosci..

[5] Lisman J, Yasuda R, Raghavachari S (2012). Mechanisms of CaMKII action in long-term potentiation. Nat. Rev. Neurosci..

[6] Giese KP, Mizuno K (2013). The roles of protein kinases in learning and memory. Learn. Mem..

[7] Herring BE, Nicoll RA (2016). Long-term potentiation: from CaMKII to AMPA receptor trafficking. Annu. Rev. Physiol..

[8] Bayer KU, Schulman H (2019). CaM kinase: still inspiring at 40. Neuron.

[9] McGuinness TL, Lai Y, Greengard P (1985). Ca2+/calmodulin-dependent protein kinase II. Isozymic forms from rat forebrain and cerebellum. J. Biol. Chem..

[10] Myers JB (2017). The CaMKII holoenzyme structure in activation-competent conformations. Nat. Commun..

[11] Lisman J, Schulman H, Cline H (2002). The molecular basis of CaMKII function in synaptic and behavioural memory. Nat. Rev. Neurosci..

[12] Bayer KU (2006). Transition from reversible to persistent binding of CaMKII to postsynaptic sites and NR2B. J. Neurosci..

[13] Zhang YP, Holbro N, Oertner TG (2008). Optical induction of plasticity at single synapses reveals input-specific accumulation of alphaCaMKII. Proc. Natl Acad. Sci. USA.

[14] Lee SJ, Escobedo-Lozoya Y, Szatmari EM, Yasuda R (2009). Activation of CaMKII in single dendritic spines during long-term potentiation. Nature.

[15] Saneyoshi T (2019). Reciprocal activation within a kinase-effector complex underlying persistence of structural LTP. Neuron.

[16] Coultrap SJ, Bayer KU (2012). CaMKII regulation in information processing and storage. Trends Neurosci..

[17] Pettit DL, Perlman S, Malinow R (1994). Potentiated transmission and prevention of further LTP by increased CaMKII activity in postsynaptic hippocampal slice neurons. Science.

[18] Lledo PM (1995). Calcium/calmodulin-dependent kinase II and long-term potentiation enhance synaptic transmission by the same mechanism. Proc. Natl Acad. Sci. USA.

[19] Jourdain P, Fukunaga K, Muller D (2003). Calcium/calmodulin-dependent protein kinase II contributes to activity-dependent filopodia growth and spine formation. J. Neurosci..

[20] Tischer D, Weiner OD (2014). Illuminating cell signalling with optogenetic tools. Nat. Rev. Mol. Cell Biol..

[21] Hayashi-Takagi A (2015). Labelling and optical erasure of synaptic memory traces in the motor cortex. Nature.

[22] Sinnen BL (2017). Optogenetic control of synaptic composition and function. Neuron.

[23] Murakoshi H (2017). Kinetics of endogenous CaMKII required for synaptic plasticity revealed by optogenetic kinase inhibitor. Neuron.

[24] Kakegawa W (2018). Optogenetic control of synaptic AMPA receptor endocytosis reveals roles of LTD in motor learning. Neuron.

[25] Hollos, P., John, J. M., Lehtonen, J. V. & Coffey, E. T. Optogenetic control of spine-head JNK reveals a role in dendritic spine regression. *eNeuro* **7**, 10.1523/ENEURO.0303-19.2019 (2020). 10.1523/ENEURO.0303-19.2019 PMC7053173 31937523

[26] Letellier, M., Lagardere, M., Tessier, B., Janovjak, H. & Thoumine, O. Optogenetic control of excitatory post-synaptic differentiation through neuroligin-1 tyrosine phosphorylation. *eLife* **9**, 10.7554/eLife.52027 (2020). 10.7554/eLife.52027 PMC7180054 32324534

[27] Wu YI (2009). A genetically encoded photoactivatable Rac controls the motility of living cells. Nature.

[28] Lu J, Zuo Y (2017). Clustered structural and functional plasticity of dendritic spines. Brain Res. Bull..

[29] Zhou XX, Fan LZ, Li P, Shen K, Lin MZ (2017). Optical control of cell signaling by single-chain photoswitchable kinases. Science.

[30] Levskaya A, Weiner OD, Lim WA, Voigt CA (2009). Spatiotemporal control of cell signalling using a light-switchable protein interaction. Nature.

[31] Chen D, Gibson ES, Kennedy MJ (2013). A light-triggered protein secretion system. J. Cell Biol..

[32] Homans RJ (2018). Two photon spectroscopy and microscopy of the fluorescent flavoprotein, iLOV. Phys. Chem. Chem. Phys..

[33] Huang S, Heikal AA, Webb WW (2002). Two-photon fluorescence spectroscopy and microscopy of NAD(P)H and flavoprotein. Biophysical J..

[34] Chao LH (2011). A mechanism for tunable autoinhibition in the structure of a human Ca2+/calmodulin- dependent kinase II holoenzyme. Cell.

[35] Halavaty AS, Moffat K (2007). N- and C-terminal flanking regions modulate light-induced signal transduction in the LOV2 domain of the blue light sensor phototropin 1 from *Avena sativa*. Biochemistry.

[36] Bhattacharyya, M. et al. Flexible linkers in CaMKII control the balance between activating and inhibitory autophosphorylation. *eLife* **9**, 10.7554/eLife.53670 (2020). 10.7554/eLife.53670 PMC7141811 32149607

[37] Yasuda R (2006). Supersensitive Ras activation in dendrites and spines revealed by two-photon fluorescence lifetime imaging. Nat. Neurosci..

[38] Shibata AC, Maebashi HK, Nakahata Y, Nabekura J, Murakoshi H (2015). Development of a molecularly evolved, highly sensitive CaMKII FRET sensor with improved expression pattern. PLoS ONE.

[39] Wang H (2016). LOVTRAP: an optogenetic system for photoinduced protein dissociation. Nat. Methods.

[40] Tanaka J (2008). Protein synthesis and neurotrophin-dependent structural plasticity of single dendritic spines. Science.

[41] Govindarajan A, Israely I, Huang SY, Tonegawa S (2011). The dendritic branch is the preferred integrative unit for protein synthesis-dependent LTP. Neuron.

[42] McCarron JG (1992). Calcium-dependent enhancement of calcium current in smooth muscle by calmodulin-dependent protein kinase II. Nature.

[43] Welsby PJ (2003). A mechanism for the direct regulation of T-type calcium channels by Ca2+/calmodulin-dependent kinase II. J. Neurosci..

[44] Jiang X (2008). Modulation of CaV2.1 channels by Ca2+/calmodulin-dependent protein kinase II bound to the C-terminal domain. Proc. Natl Acad. Sci. USA.

[45] Chen TW (2013). Ultrasensitive fluorescent proteins for imaging neuronal activity. Nature.

[46] Kopec CD, Li B, Wei W, Boehm J, Malinow R (2006). Glutamate receptor exocytosis and spine enlargement during chemically induced long-term potentiation. J. Neurosci..

[47] Makino H, Malinow R (2011). Compartmentalized versus global synaptic plasticity on dendrites controlled by experience. Neuron.

[48] Murakoshi H, Wang H, Yasuda R (2011). Local, persistent activation of Rho GTPases during plasticity of single dendritic spines. Nature.

[49] Hedrick NG (2016). Rho GTPase complementation underlies BDNF-dependent homo- and heterosynaptic plasticity. Nature.

[50] Murakoshi H, Shibata ACE (2017). ShadowY: a dark yellow fluorescent protein for FLIM-based FRET measurement. Sci. Rep..

[51] Sells MA (1997). Human p21-activated kinase (Pak1) regulates actin organization in mammalian cells. Curr. Biol..

[52] Kleindienst T, Winnubst J, Roth-Alpermann C, Bonhoeffer T, Lohmann C (2011). Activity-dependent clustering of functional synaptic inputs on developing hippocampal dendrites. Neuron.

[53] Takahashi N (2012). Locally synchronized synaptic inputs. Science.

[54] Cichon J, Gan WB (2015). Branch-specific dendritic Ca2+ spikes cause persistent synaptic plasticity. Nature.

[55] Berggard T (2006). 140 mouse brain proteins identified by Ca2+-calmodulin affinity chromatography and tandem mass spectrometry. J. Proteome Res..

[56] Otmakhov N, Lisman J (2012). Measuring CaMKII concentration in dendritic spines. J. Neurosci. Methods.

[57] Fino E (2009). RuBi-glutamate: two-photon and visible-light photoactivation of neurons and dendritic spines. Front. Neural Circuits.

[58] Matsuzaki M (2001). Dendritic spine geometry is critical for AMPA receptor expression in hippocampal CA1 pyramidal neurons. Nat. Neurosci..

[59] Palfi D (2018). High efficiency two-photon uncaging coupled by the correction of spontaneous hydrolysis. Org. Biomol. Chem..

[60] Ryan XP (2005). The Rho-specific GEF Lfc interacts with neurabin and spinophilin to regulate dendritic spine morphology. Neuron.

[61] Araki Y, Zeng M, Zhang M, Huganir RL (2015). Rapid dispersion of SynGAP from synaptic spines triggers AMPA receptor insertion and spine enlargement during LTP. Neuron.

[62] Harvey CD, Yasuda R, Zhong H, Svoboda K (2008). The spread of Ras activity triggered by activation of a single dendritic spine. Science.

[63] Shaner NC (2004). Improved monomeric red, orange and yellow fluorescent proteins derived from *Discosoma* sp. red fluorescent protein. Nat. Biotechnol..

[64] Donnelly ML (2001). Analysis of the aphthovirus 2A/2B polyprotein ‘cleavage’ mechanism indicates not a proteolytic reaction, but a novel translational effect: a putative ribosomal ‘skip’. J. Gen. Virol..

[65] Zacharias DA, Violin JD, Newton AC, Tsien RY (2002). Partitioning of lipid-modified monomeric GFPs into membrane microdomains of live cells. Science.

[66] Murakoshi H, Shibata AC, Nakahata Y, Nabekura J (2015). A dark green fluorescent protein as an acceptor for measurement of Forster resonance energy transfer. Sci. Rep..

[67] Lock M (2010). Rapid, simple, and versatile manufacturing of recombinant adeno-associated viral vectors at scale. Hum. Gene Ther..

[68] Stoppini L, Buchs PA, Muller D (1991). A simple method for organotypic cultures of nervous tissue. J. Neurosci. Methods.

[69] Pologruto TA, Sabatini BL, Svoboda K (2003). ScanImage: flexible software for operating laser scanning microscopes. Biomed. Eng. Online.

[70] Murakoshi, H. & Shibata, A. C. Optogenetic Imaging of Protein Activity Using Two-Photon Fluorescence Lifetime Imaging Microscopy. in *Optogenetics* (eds Yawo, H., Kandori, H. & Koizumi, A.) Ch. 12, 185–197 (Springer Japan, 2015).

