# Supplementary material for: Photoactivatable CaMKII induces synaptic plasticity in single synapses
Source: Nat Commun. 2021 Feb 2;12:751. doi: 10.1038/s41467-021-21025-6 (PMC7854602; doi:10.1038/s41467-021-21025-6)
Supplement: Supplementary file 1 — Supplementary Information [file 41467_2021_21025_MOESM1_ESM.pdf]

Supplementary Information

Photoactivatable CaMKII induces synaptic plasticity in single synapses

Akihiro C. E. Shibata, Hiromi H. Ueda, Kei Eto, Maki Onda, Aiko Sato, Tatsuko Ohba, Junichi Nabekura, and Hideji Murakoshi

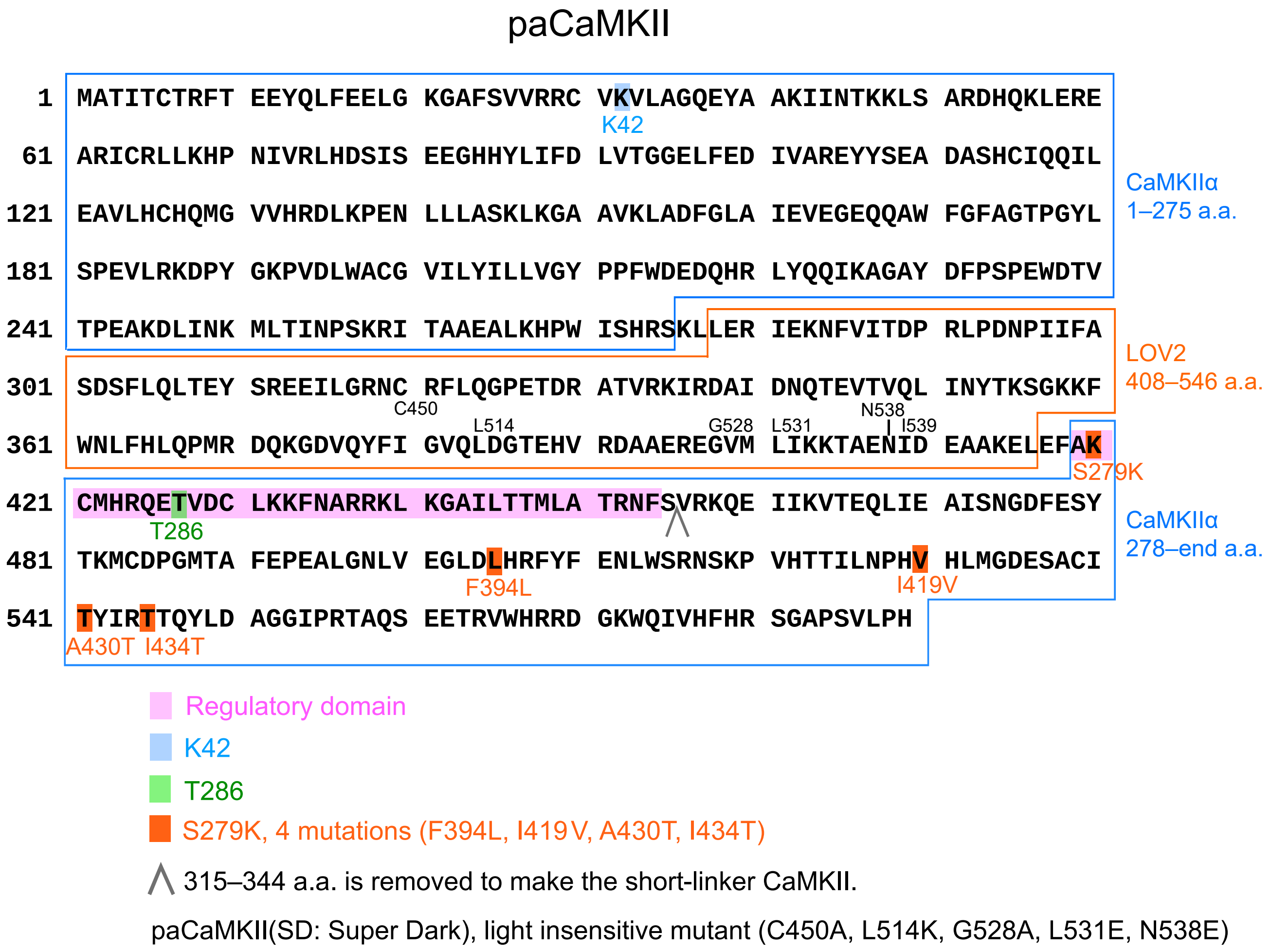

Supplementary Figure 1: Sequence of paCaMKII.

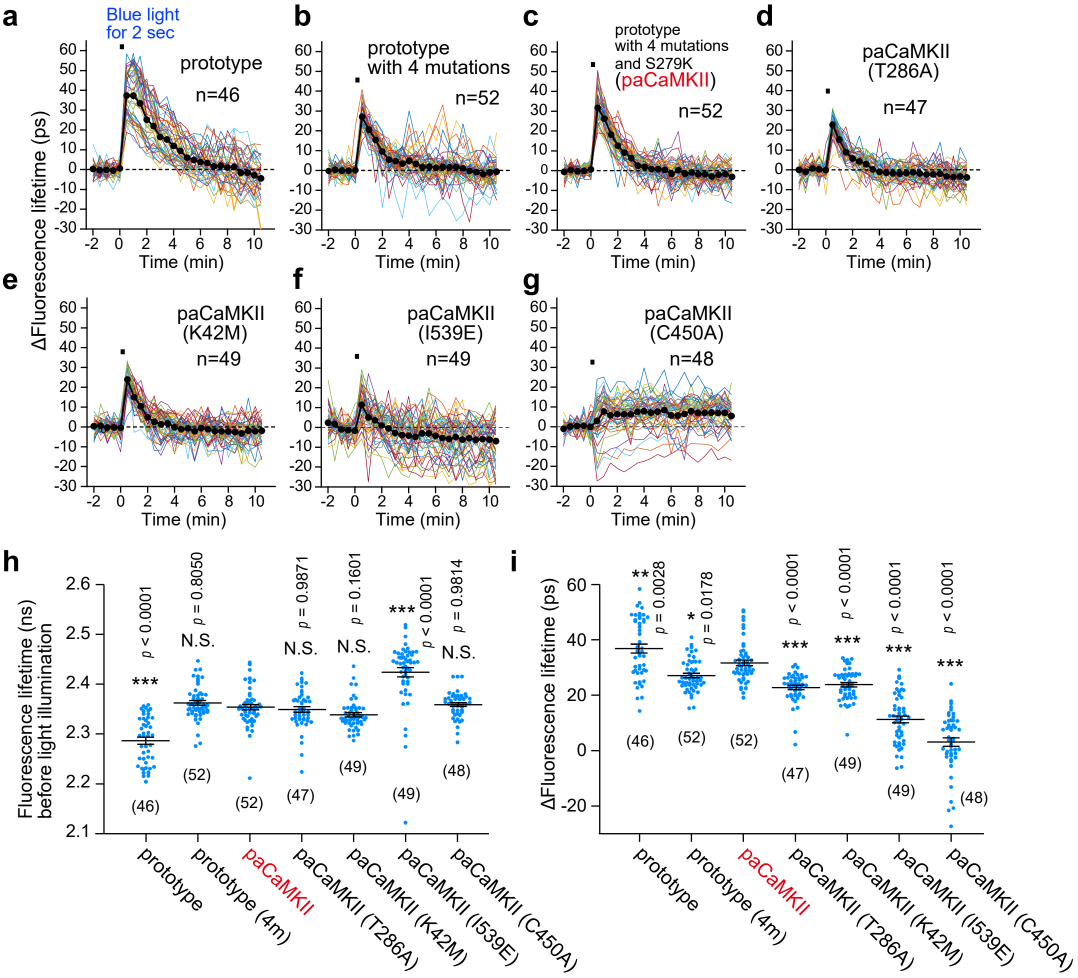

Supplementary Figure 2: Structural characterization of paCaMKII.

(**a–g**) The lifetime change (conformational change) of mEGFP-paCaMKII-ShadowG in individual HeLa cells after blue light illumination. A prototype (a), with four mutations (4m, F394L/I419V/A430T/I434T) (b), with four mutations and S279K (i.e., paCaMKII) (c, the same plot presented in Fig. 1g), autophosphorylation-deficient mutant (T286A) (d), Kinase-dead mutant (K42M) (e), constitutively open LOV2 mutant (I539E) (f), and light-insensitive LOV2 mutant (C450A) (g, the same plot presented in Fig. 1h). Colored lines represent the response signals from individual cells, and the black circles indicate an averaged timecourse. The data are presented as mean ± SEM. The number of samples (n) is shown in the respective panels.

(**h, i**) The fluorescence lifetime of individual cells before blue light illumination (h) and the fluorescence lifetime changes at 30 sec (immediately after blue light illumination) (i) were quantified using the data presented in (a–g), respectively. The number of samples (n) is indicated in the respective panels. Black lines represent the mean values +/- SEM. ***$p < 0.001$; **$p < 0.01$; *$p < 0.05$; N.S. not significant; one-way ANOVA followed by Dunnet's post-hoc test. The results of statistical tests are shown only for the comparison with paCaMKII and the rest of the mutants. Source data are provided as a Source Data file.

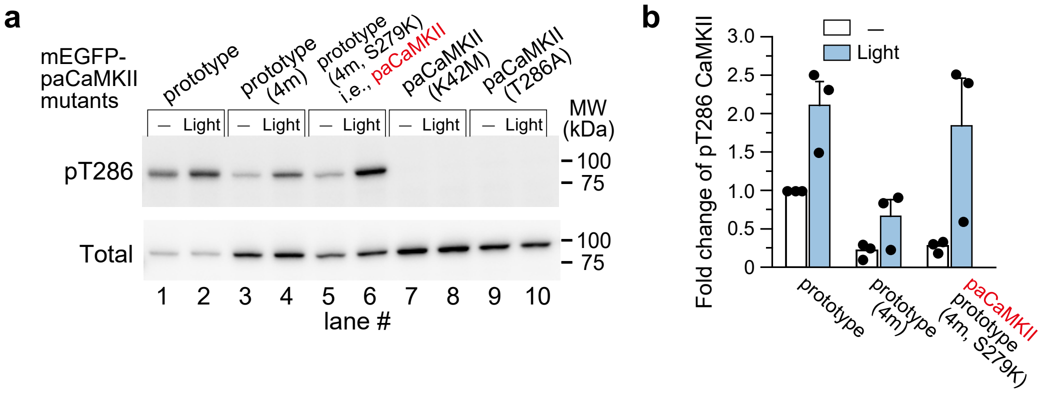

Supplementary Figure 3: Biochemical assay of paCaMKII.

(**a**) Light-dependent Thr286 autophosphorylation of paCaMKII. mEGFP-paCaMKII was expressed in HeLa cells by lipofection. Samples were continuously illuminated for 2 min with blue LED light at 3 mW cm^{-2}. Prototype paCaMKII phosphorylation (lane #1, #2), and with four mutations (4m, F394L/I419V/A430T/I434T) (lane #3, #4). Prototype with four mutations and S279K (i.e., paCaMKII) autophosphorylation (lane #5, #6). Both the kinase-dead mutant (K42M) and autophosphorylation-deficient mutant (T286A) failed to autophosphorylate after blue light exposure (lane #7–10).

(**b**) Quantification of paCaMKII autophosphorylation. The band intensities of p286 were divided by those of total and normalized with lane#1. The data are presented as mean values +/- SEM. n=3 for independent experiments. Source data are provided as a Source Data file.

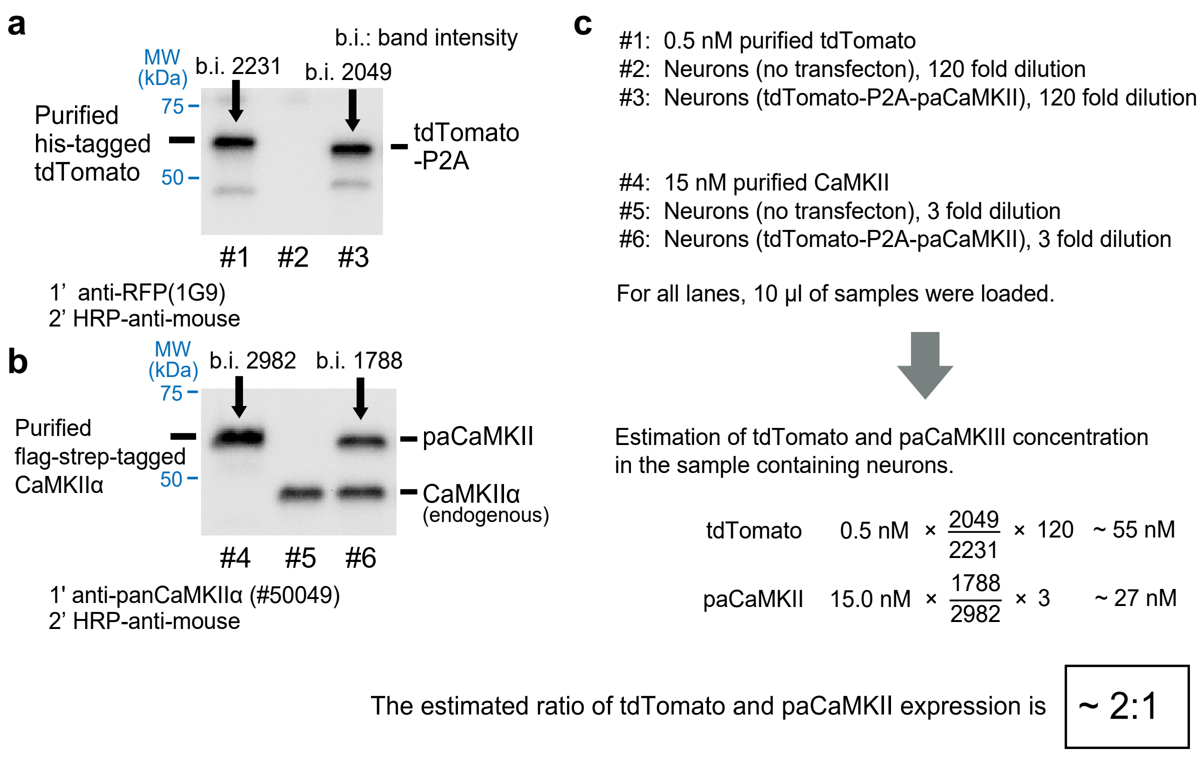

Supplementary Figure 4: Estimation of the tdTomato and paCaMKII expression ratio.

(**a, b**) Western blots of dissociated hippocampal neurons infected with AAV-DJ encoding CaMKII promotor-tdTomato-P2A-paCaMKII, and the known concentration of purified tdTomato (a) and CaMKII (b).

(**c**) To estimate the ratio of tdTomato and paCaMKII expressed via the P2A sequence, we first measured tdTomato and paCaMKII concentrations in the neuronal sample by comparing the band intensity of purified proteins. The estimated ratio of tdTomato and paCaMKII is approximately 2:1. While the P2A sequence is believed to express two or more proteins equally, our results suggest that tdTomato expression is higher than paCaMKII expression. This may be due to the more efficient translation of tdTomato than paCaMKII or/and more rapid degradation of paCaMKII than tdTomato. Thus, two scales are used in Fig. 3h and i. The experiments were reproduced once with similar results. Source data (i.e., the band intensities) are provided in the figure.

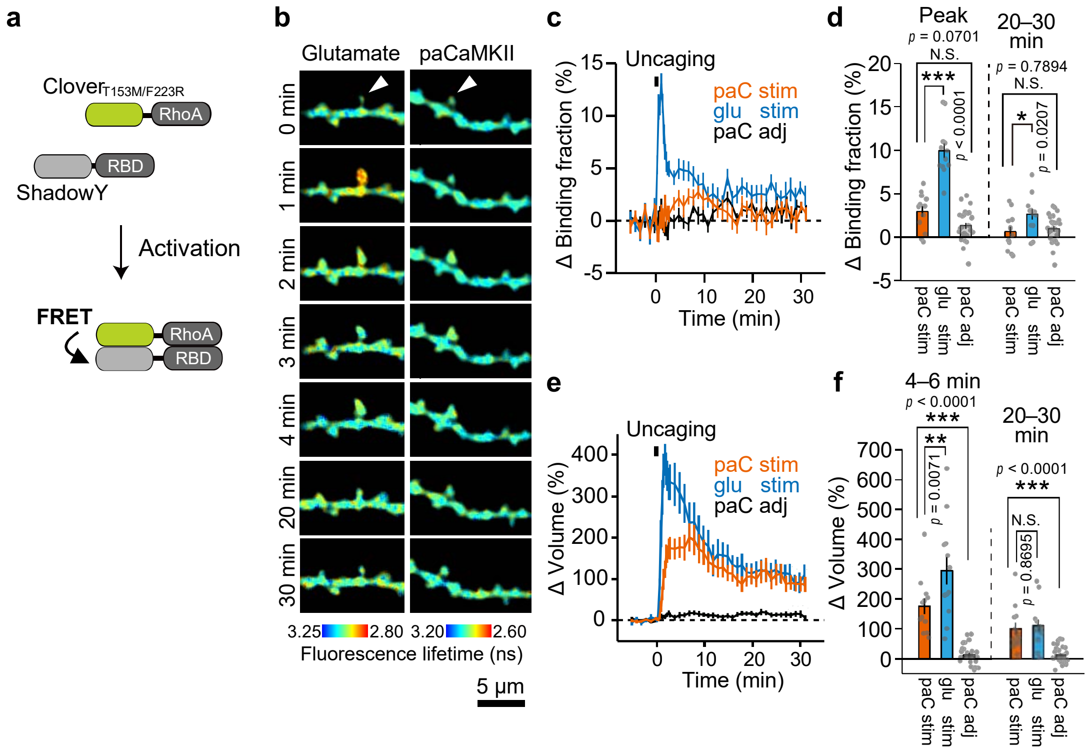

Supplementary Figure 5: Spatiotemporal dynamics of RhoA activation upon single-spine paCaMKII activation.

(**a**) Schematic of RhoA FRET sensor activation.

(**b**) Fluorescence lifetime images of RhoA activity during the induction of sLTP by 2-photon glutamate uncaging (left) and paCaMKII uncaging (right). RhoA FRET sensor and tdTomato-P2A-paCaMKII were co-expressed in hippocampal CA1 neurons in a cultured slice. Two-photon excitation at 1010 nm was used to excite $Clover_{T154M/F223R}$. Two-photon excitation at 720 nm (30 trains, 0.5 Hz, 6 ms duration/pulse, 6 mW) and 820 nm (30 trains, 0.5 Hz, 40 ms duration/pulse, 4 mW) was used to uncage MNI-caged glutamate and paCaMKII, respectively. White arrows indicate the stimulated spine. The warmer color indicates higher RhoA activity. Scale bar, 5 µm.

(**c, e**) Averaged timecourse of the binding fraction change of $Clover_{T154M/F223R}$-RhoA (RhoA activation) (c) and spine volume change (e) upon glutamate uncaging (glu stim), paCaMKII activation (paC stim, stimulated spines; paC adj; adjacent spines, 2-10 µm of space from the stimulated spines), and no light stimulation (paC no light). n(spines/neurons) = 12/6 paC stim, 27/6 paC adj, 17/5 paC no light, and 12/7 glu stim.

(**d, f**) Quantification of transient (five-point rolling average at peak) (d, left) and sustained (averaged over 20–30 min) (d, right) binding fraction change. And quantification of spine volume change averaged over 4–6 min (f, left) and averaged over 20–30 min (f, right). The same data set with c and e were used for quantification. n(spines/neurons) is the same as in fig (c) and (e), respectively. The data are presented as mean ± SEM; ****p* < 0.001; ***p* < 0.01; **p* < 0.05; N.S. not significant; one-way ANOVA followed by Dunnet's post-hoc test. Source data are provided as a Source Data file.

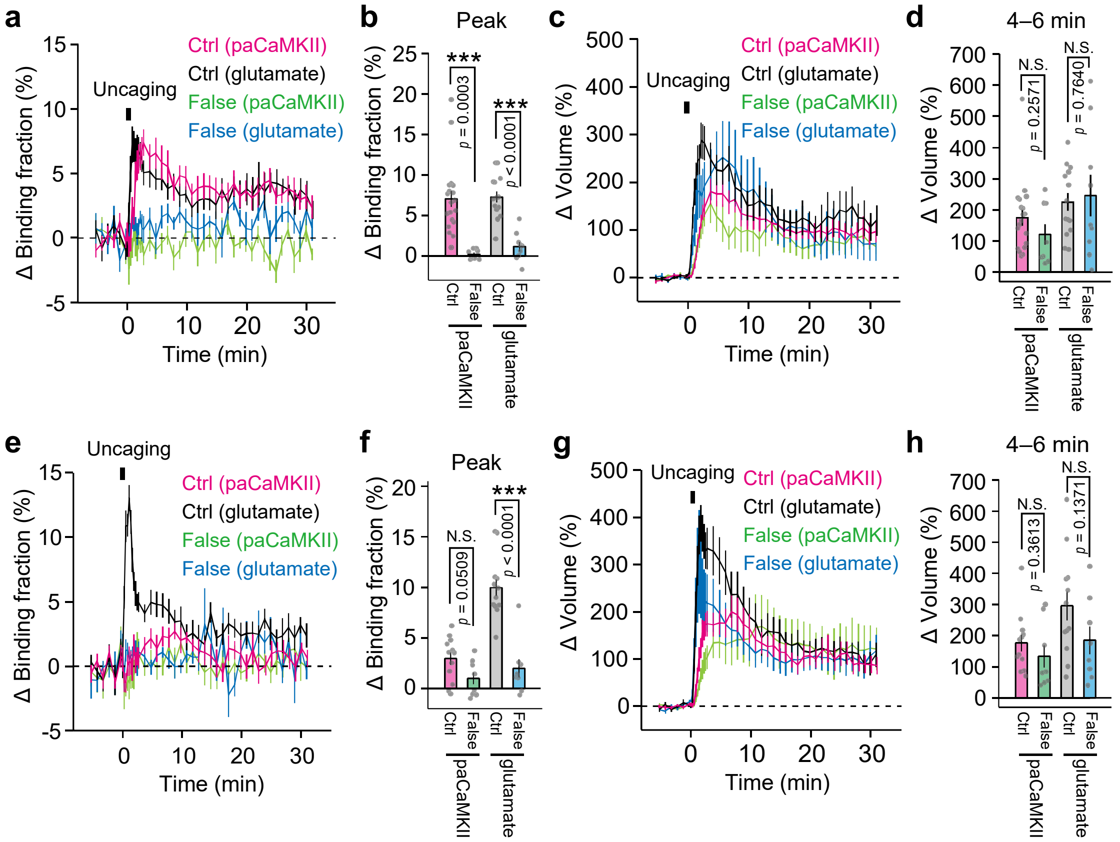

Supplementary Figure 6: Fluorescence lifetime change of Clover$_{T154M/F223R}$ -Cdc42 or -RhoA paired with false acceptor during sLTP.

(**a, e**) Averaged timecourse of Cdc42 (a) and RhoA (e) activation measured as a change in the fraction of Clover$_{T154M/F223R}$-Cdc42 or -RhoA bound ShadowY-CBD (H83L, H86L) upon paCaMKII activation (False, paCaMKII) or glutamate uncaging (False, glutamate) uncaging. For comparison, the timecourses of the right Cdc42 and RhoA sensor (Ctrl with paCaMKII or glutamate) from Fig. 6c and S5c are also plotted in a and e, respectively. For the Cdc42 experiment (a–d), n(spines/neurons) = 8/6 false (paCaMKII), 9/6 false (glutamate). For the RhoA experiment (e–h), n(spines/neurons) = 8/6 false (paCaMKII), 8/8 false (glutamate).

(**b, f**) Quantification of transient binding fraction change (five-point rolling average at peak) of Cdc42 (b) and RhoA (f). The same data set with (a) and (e) were used for quantification, respectively. n(spines/neurons) is the same as in fig a and e, respectively.

(**c, g**) Averaged timecourse of spine volume change in the same experiments as in (a) and (e), respectively. For comparison, the timecourses of the right Cdc42 and RhoA sensor (Ctrl with paCaMKII or glutamate) from Fig. 6e and S5e are also plotted in a and e, respectively.

(**d, h**) Quantification of spine volume change averaged over 4–6 min of Cdc42 (d) and RhoA (h). The same data set with c and g were used for quantification. n(spines/neurons) is the same as in fig (c) and (g), respectively.

All data are presented as mean ± SEM; ***$p < 0.001$; **$p < 0.01$; *$p < 0.05$; N.S. not significant; two-tailed unpaired *t* test (figs. b, d, f, h). Source data are provided as a Source Data file.

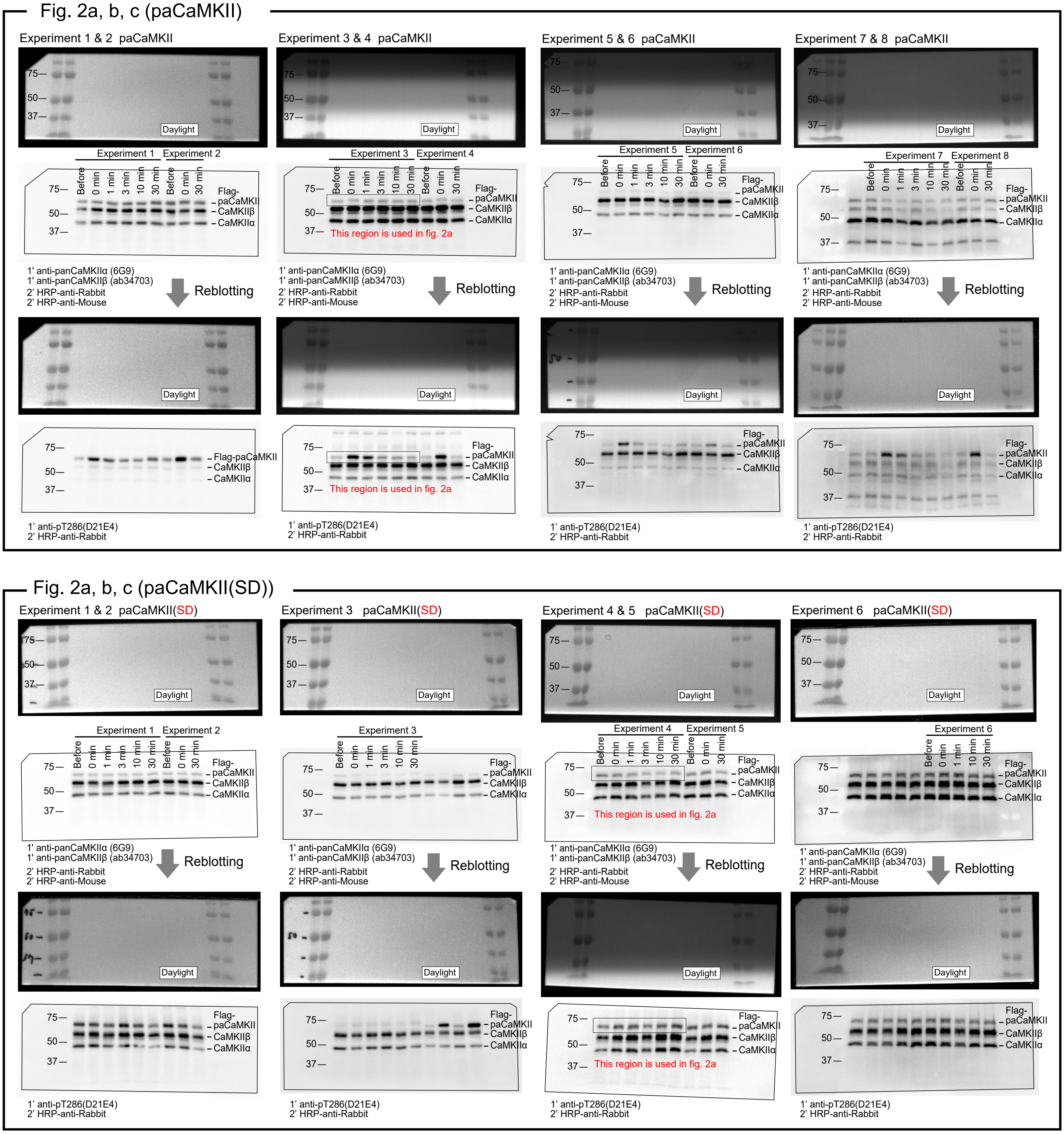

Supplementary Figure 7: Uncropped images of blots

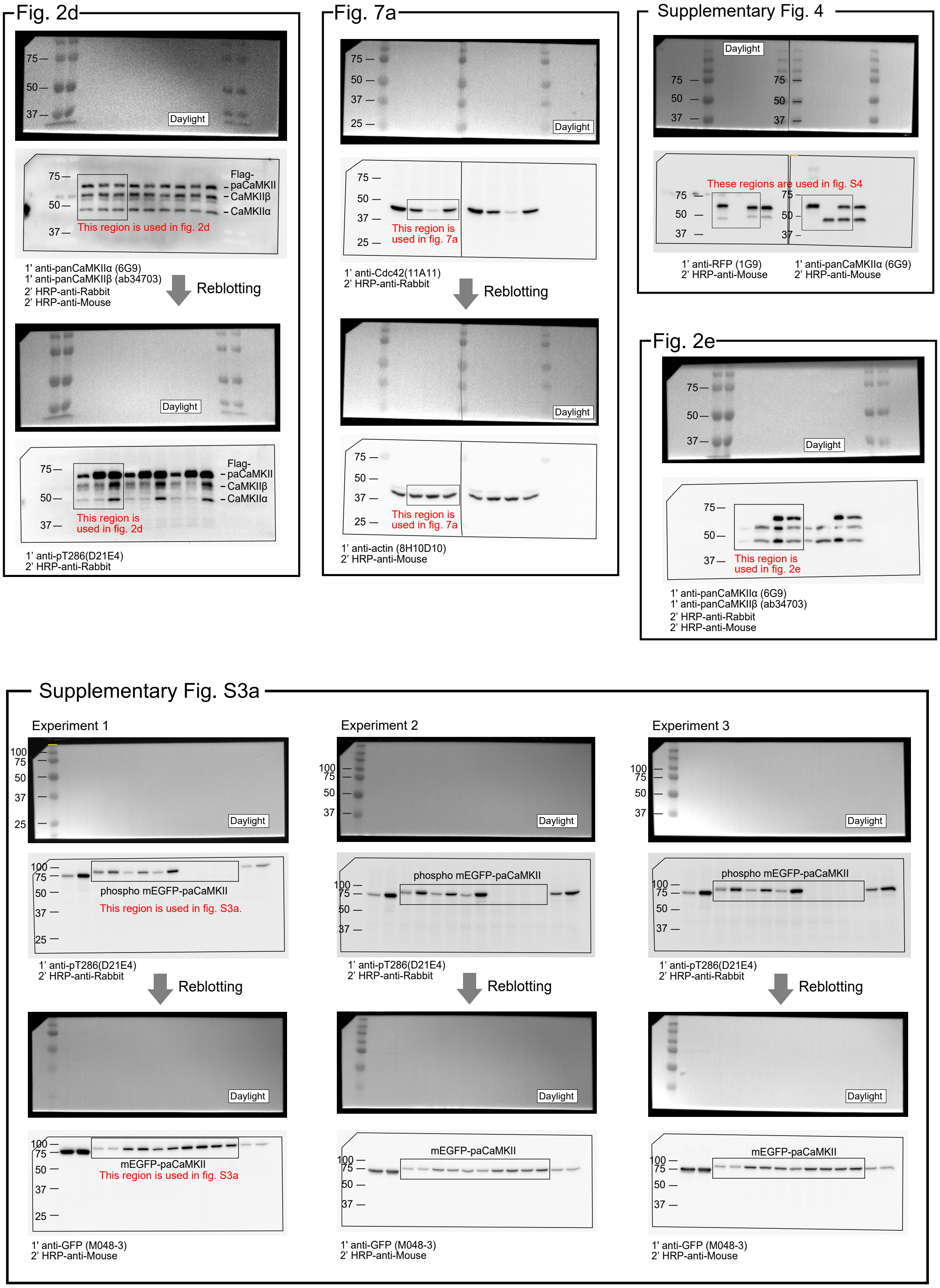

Supplementary Figure 7: Uncropped images of blots
