# Supplementary material for: Photoactivatable CaMKII induces synaptic plasticity in single synapses
Source: Nat Commun. 2021 Feb 2;12:751. doi: 10.1038/s41467-021-21025-6 (PMC7854602; doi:10.1038/s41467-021-21025-6)
Supplement: Supplementary file 3 — Reporting summary [file 41467_2021_21025_MOESM3_ESM.pdf]

nature research

Corresponding author(s): Hideji Murakoshi

Last updated by author(s): Nov 25, 2020

Reporting Summary

Nature Research wishes to improve the reproducibility of the work that we publish. This form provides structure for consistency and transparency in reporting. For further information on Nature Research policies, see Authors & Referees and the Editorial Policy Checklist .

Statistics

For all statistical analyses, confirm that the following items are present in the figure legend, table legend, main text, or Methods section.

n/a	Confirmed	
☐	☒	The exact sample size (*n*) for each experimental group/condition, given as a discrete number and unit of measurement
☐	☒	A statement on whether measurements were taken from distinct samples or whether the same sample was measured repeatedly
☐	☒	The statistical test(s) used AND whether they are one- or two-sided<br>*Only common tests should be described solely by name; describe more complex techniques in the Methods section.*
☒	☐	A description of all covariates tested
☒	☐	A description of any assumptions or corrections, such as tests of normality and adjustment for multiple comparisons
☐	☒	A full description of the statistical parameters including central tendency (e.g. means) or other basic estimates (e.g. regression coefficient) AND variation (e.g. standard deviation) or associated estimates of uncertainty (e.g. confidence intervals)
☐	☒	For null hypothesis testing, the test statistic (e.g. *F*, *t*, *r*) with confidence intervals, effect sizes, degrees of freedom and *P* value noted<br>*Give P values as exact values whenever suitable.*
☒	☐	For Bayesian analysis, information on the choice of priors and Markov chain Monte Carlo settings
☒	☐	For hierarchical and complex designs, identification of the appropriate level for tests and full reporting of outcomes
☒	☐	Estimates of effect sizes (e.g. Cohen's *d*, Pearson's *r*), indicating how they were calculated

Our web collection on statistics for biologists contains articles on many of the points above.

Software and code

Policy information about availability of computer code

Data collection	NA
Data analysis	NA

For manuscripts utilizing custom algorithms or software that are central to the research but not yet described in published literature, software must be made available to editors/reviewers. We strongly encourage code deposition in a community repository (e.g. GitHub). See the Nature Research guidelines for submitting code & software for further information.

Data

Policy information about availability of data

All manuscripts must include a data availability statement. This statement should provide the following information, where applicable:

- Accession codes, unique identifiers, or web links for publicly available datasets
- A list of figures that have associated raw data
- A description of any restrictions on data availability

The data generated and analyzed during the current study are included in this published article and its supplementary information file. Datasets are available from the corresponding author on request.

Field-specific reporting

Please select the one below that is the best fit for your research. If you are not sure, read the appropriate sections before making your selection.

☒ Life sciences ☐ Behavioural & social sciences ☐ Ecological, evolutionary & environmental sciences

For a reference copy of the document with all sections, see nature.com/documents/nr-reporting-summary-flat.pdf

Life sciences study design

All studies must disclose on these points even when the disclosure is negative.

Sample size	Sample size for each experiment is indicated in the figure legend for each experiment. The sample size was chosen based on previous experience for each experiment to yield high power to detect specific effects. No statistical methods were used to predetermine sample size.
Data exclusions	No data were exclued from the analysis.
Replication	All experimental findings were reliably reproducible.
Randomization	Each samples were randomly allocated into experimental groups.
Blinding	For all experiments, the investigators were blind to the experimental groups during the analyses.

Reporting for specific materials, systems and methods

We require information from authors about some types of materials, experimental systems and methods used in many studies. Here, indicate whether each material, system or method listed is relevant to your study. If you are not sure if a list item applies to your research, read the appropriate section before selecting a response.

Materials & experimental systems

n/a	Involved in the study	
☐	☒	Antibodies
☐	☒	Eukaryotic cell lines
☒	☐	Palaeontology
☐	☒	Animals and other organisms
☒	☐	Human research participants
☒	☐	Clinical data

Methods

n/a	Involved in the study	
☒	☐	ChIP-seq
☒	☐	Flow cytometry
☒	☐	MRI-based neuroimaging

Antibodies

Antibodies used	The antibodies used: anti-Phospho-CaMKII (Thr286) (D21E4, Cell Signaling Technology; Danvers, MA, USA); anti-CaMKIIα (6G9; Cell Signaling Technology); anti-CaMKIIβ (ab34703; Abcam); anti-GFP (M048-3, MBL; Woburn, MA, USA); anti-Cdc42 (11A11; Cell Signaling Technology); anti-β-Actin (8H10D10; Cell Signaling Technology); HRP-anti-mouse and -rabbit (Jackson Laboratory; Bar Harbor, ME, USA);anti-RFP (1G9, MBL; Woburn, MA, USA).
Validation	All antibodies used in our manuscript were validated by the manufactures or validated in the manuscript.

Eukaryotic cell lines

Policy information about cell lines

Cell line source(s)	HeLa, HEK293
Authentication	None of the cell lines used were authenticated.
Mycoplasma contamination	The cell lines were not tested for mycoplasma contamination.
Commonly misidentified lines (See ICLAC register)	NA

Animals and other organisms

Policy information about studies involving animals; ARRIVE guidelines recommended for reporting animal research

Laboratory animals	All dissociated cultures were prepared from C57BL/6N mice (SLC). All slice cultures were prepared from Wistar rats (SLC or Charles River). This study used dissociated and slice cultures from both male and female pups. For in vivo imaging, the experiments were performed using male and female adult (2 months) C57BL/6N mice (Charles River).
Wild animals	NA

October 2018

Field-collected samples	NA
Ethics oversight	All animal procedures were approved by the National Institutes of Natural Sciences Animal Care and Use Committee and were performed under the relevant guidelines and regulations.

Note that full information on the approval of the study protocol must also be provided in the manuscript.
