# Supplementary material for: Photoactivatable CaMKII induces synaptic plasticity in single synapses
Source: Nat Commun. 2021 Feb 2;12:751. doi: 10.1038/s41467-021-21025-6 (PMC7854602; doi:10.1038/s41467-021-21025-6)
Supplement: Supplementary file 2 — Peer Review File [file 41467_2021_21025_MOESM2_ESM.pdf]

REVIEWER COMMENTS

Reviewer #1 (Remarks to the Author):

This study by Shibata et al introduces an elegant and highly sophisticated new tool for research on CaMKII and synaptic plasticity: a photo-activatable paCaMKII. A long series of prominent previous pharmacological and genetic studies ha established the necessity of CaMKII in LTP over the last 30 years. Using their novel tool, Shibata et al here for the first time convincingly test and demonstrate also sufficiency of CaMKII activation (using a realistic activation pattern). As LTP is thought to underly higher brain functions such as learning, memory, and cognition, these findings should be of high interest to a broad audience. This is further enhanced by the facts that the study design is beautiful, the experiments are rigorous, the scope is spanning from molecular mechanisms to in vivo analysis, and the writing is generally easily accessible.
While I have a rather lengthy list of comments, they are all very minor and do not require any additional experimentation.

Main comment:
The mechanism of autophosphorylation of paCaMKII deserves a little more discussion (because this is a new tool; because autophosphorylation is important for inducing plasticity; and because some quite interesting data are presented on it that may deserve some more attention). First of all, for endogenous CaMKII, T286 autophosphorylation occurs as a reaction between two subunits within a holoenzyme, and both subunits require binding of Ca2+/CaM. The data shown here indicate that photo-activation of paCaMKII can substitute for Ca2+/CaM not only on the subunit acting as kinase, but also on the subunit acting as substrate. This, in a way, is not unexpected, as the photoactivation should indeed make T286 accessible as substrate (but it would be good to explicitly mention this). However, the data also indicate that paCaMKII co-assembles with endogenous CaMKII into heteromeric holoenzymes. In response to Ca2+-stimuli, all subunits get autophosphorylated; in response to light, only the paCaMKII subunits get autophosphorylated. This is great (and functionally important for this tool, as shown later), but it is slightly surprising that assembly into heteromeric holoenzyme does not seem to reduce the light-induced autophosphorylation of the paCaMKII (because addition of endogenous CaMKII should decrease the chance that paCaMKII assemble as neighbors to phosphorylate each other). This maybe explained by insertion of the LOV2 domain inducing a "wider reach", i.e. beyond just the immediate neighboring subunit. But this would be worth discussing, perhaps also in context with estimates of the expression level estimates of endogenous CaMKII versus paCaMKII (to estimate how many paCaMKII subunits would be expected per holoenzyme).

Further minor comments:
L38: change "leading to LTP" to "which can lead to LTP". (Overall the start of the introduction is nicely written to enable understanding by a wide non-expert audience; but this statement could be misinterpreted to indicate glutamate release always leads to LTP).

L39: consider noting NMDARs as "NMDA-type glutamate receptors" (similarly as done a couple of lines down for AMPARs).

L46 and down: Many useful and relevant review articles on CaMKII are cited (including from Bayer and from Schulman), however, a more recent major review of CaMKII is missing (Bayer and Schulman, 2019 in Neuron).

L60: consider rephrasing "researchers have developed".

Fig1a-c is excellent for illustrating how the paCaMKII construct works. However, to the non-expert, it also suggests that CaMKII does exist to a significant extent also as monomeric subunit (which it does not). Thus, it might help to insert a panel that shows the CaMKII holoenzyme (likely as Fig1a or b).

L92: "with the human beta-7 linker" is a bit misleading, I believe. To my understanding, the minor

beta-7 isoform completely lacks a linker region. Thus, the paCaMKII construct has a LOV2 domain inserted and the linker region deleted. I believe there may actually be good rationale for deleting the linker region (which is relatively short in alpha anyway), such as that it might decrease the radius of how far the activated kinase domain can move away from the central hub (thereby compensating for the likely increase in radius introduced by insertion of the LOV2 domain). To make this point, inserting a panel with the holoenzyme in Fig1 may help (as also suggested above). For holoenzyme structure and radius considerations see also Myers et al 2017 in Nature Communications.

L108: It might be helpful to note here that the association domain mutations in the optimized paCaMKII do not affect holoenzyme formation (as indicated later in Fig1g).

(L136: consider rewording the sentence.)

L140: Indicate duration of expression of the His-tagged paCaMKII here (especially as it is also unclear from the methods, which indicate infection a div 9-11 and harvest at div 12-15; so it could be 1 to 6 days for this experiment).

L141: reword, as there does not seem to be any evidence that phosphorylation at T286 is "critical" for activation of paCaMKII (even though it is later shown that it is indeed critical for the function of paCaMKII in plasticity).

L173: To my understanding, CaMKII interaction with L-type channels was described even before T- and P/Q-type interactions.

Fig.4d: Legend indicates paCaMKII activation for the sample traces (the measure one would want to see), but in the figure, "glutamate uncaging" is indicated instead.

L257+262: "This difference suggests that other signaling pathways beside CaMKII pathway may be involved in the initial phase" and "These findings suggest that CaMKII-Cdc42 might be a major pathway for inducing sLTP." Consider rewriting in terms of sufficiency versus necessity of CaMKII. (CaMKII has been shown to be necessary for activation of both cdc42 and rhoA. These data indicated that it is also sufficient for activation of cdc42 but not rhoA). The current writing might be mis-interpreted to suggest that the CaMKII is involved only in activation of cdc42 but not rhoA. Similar for the discussion.

Fig. 7: title of the legend indicates in vitro and in vivo, but the data shown in this figure are exclusively in vivo imaging (the in vitro was shown in previous figures).

L315: Consider rewriting, since in the current form, this is an overstatement. One can "separate" CaMKII from other Ca2+-activated processes by inhibition, to test necessity; the point is that this new approach allows direct activation to directly test also sufficiency. Perhaps consider this comparison also for the abstract, i.e. contrasting the long-known necessity of CaMKII in LTP with the here-shown sufficiency of CaMKII activation.

Reviewer #2 (Remarks to the Author):

In this manuscript Murakoshi et al. report the development of an optogenetic tool to control the Ca^{2+}/calmodulin-dependent protein kinase II (PACaMKII). The authors use the tool to study several aspects of synaptic plasticity involving CaMKII, including differential signaling in single vs. clustered spines and the requirement for Cdc42 activation in robust structural long-term potentiation. Both the tool and the biological studies are significant advances. However, concerns remain re both:

Major comments:

• Line 88: LOV's recovery rate can be tuned with point mutations. This is also true for other systems.
• Line 99-101: The authors provide very few details on one of the steps used to validate the

optogenetic tool (2pFLIM-FRET) and the reader is referred to a supplementary figure. Because appropriate validation of the tool underpins all the biological conclusions, I suggest the authors expand more on this in the text and move some of the data in Fig S1 to the main text.

- Fig S1 j): I539E (lit mutant) has a significantly lower lifetime than wild type PaCaMKII. Shouldn't this mutant have identical or higher lifetime than wild type PaCaMKII?
- Line 112 and Supplementary Fig. 2: "(…) suppressed background kinase activity in the dark condition (…)". This is not clear when comparing prototype with prototype (4m); both light and dark bars seem to decrease and the ratio seems identical. In Figure S2, it is unclear what "divided by EGFP" means when image a) only displays phosphorylated and total mEGFP-PACaMKII. How do the authors justify the clear difference in prototype and prototype (4m) being absent in Fig S1 j, where no statistical difference is found for the FLIM assay, and what is described as a "reduced light-dependent response variability" which does not seem to hold true in the blotting data for the S279K mutation?
- Fig 1d): no loading controls are provided, preventing direct comparison of number of cells and expression levels. I would suggest the authors include the full, uncropped blots as Supplementary Material for these and other Western Blotting data.
- Lines 140-143, Figure 1F: Doesn't the data from NMDA stimulation in the dark show that this tool still responds to upstream signaling, i.e. is not solely controlled by light? This indicates that expression perturbs resting CaMKII signaling, so at the very least expression level should be carefully controlled and effects in the dark should be gauged as a function of expression. This should be discussed in the text.
- Lines 169-170 and Fig2 h and i: The authors' method to estimate the concentration of PaCaMKII is problematic. Because of the p2a ribosomal skip sequence, expression of tdTomato and PaCaMKII will be identical but degradation rates can differ massively; the concentration of the two species can't simply be assumed to be identical. Furthermore, the linear fits are not useful on what seems to be a more stochastic than trended distribution. Lastly, in the Materials and Methods pertaining to this experiment, I would suggest the authors describe approximate volume for apical dendrites as opposed to "thick apical dendrites".
- Figure 3a: Images provided for GCaMP imaging are particularly noisy when compared to all other images in the document. Do the authors have an explanation for this?
- Figure 3b: This panel would benefit from control spine measurements near the PA and glutamate uncaging as done elsewhere in Figure 2.
- Lines 292-298 and Fig 6: Authors employ a single shRNA to knockdown Cdc42; I would suggest validation with a second shRNA to confirm these results, particularly because the control seems to have an effect when compared to knockdown and rescue. This could also stem from a lack of the wild type data to directly compare these effects.
- Two photon considerations: I have several concerns regarding these experiments:

o Lines 84-88: Comparison of two-photon absorption cross section values with FAD is only relevant for the CRY2 system and does not address others. References provided refer to free FMN (not in a protein). Reference 70 would be more appropriate here; providing actual values would be beneficial too.

o Lines 337-339: authors cite values of ~0.2 GM at 800–900 nm for iLOV, yet the reference provides values ranging from ~1 to that value. The reference provided for MNI-glutamate TPA cross section only provides a single value at a single wavelength, which complicates this comparison. Do the authors have a reference for a full TPA spectrum?

o Cross-photoactivation by label and biosensor imaging: Even though iLOV TPA seems to mostly drop close to zero at >950 nm, studies for closely related FMN-binding proteins (e.g. Nanna et al. 2014) show higher TPA cross section values. Determining particularly low TPA cross section values is prone to large variations. Furthermore, a small but noticeable effect is visible on volume change and binding fraction in control spines (Fig 2 b), Fig 5 c and e). This should at least be addressed in the text; ideally the authors should show that wavelengths of ≥1000 nm do not photoactivate PaCaMKII, extend their period before photoactivation, and/or perform a control experiment where cells containing PaCaMKII and labels (tdTomato and sensors) are imaged without photoactivation pulses.

Other comments:

- Line 82: The exact size depends on which LOV protein is chosen, I would suggest authors change this to ~140 a.a.
- Line 389: I believe the authors meant "directly identify" and not "directory identifies".

• Fig1: I would suggest the authors change their naming of main experiment “Ctrl” to “Stim” as used elsewhere, so the reader does not think it is a control.
• Materials and Methods: “Note that equation (1) can be applied to spherical structure Since the dendrite cannot be assumed as spherical, Rdendrite_surface is an approximate calculation.” I would suggest the authors reword this phrase.
• Can the authors explain why they started with mScarlet but switched to tdTomato for most of the experiments?
• I would suggest significance values be provided in each image where statistical analysis is performed (and what test performed).

Response to reviewers

We first would like to thank the reviewers for their valuable comments and suggestions. In response to the reviewers' comments, we have made the point-by-point responses in the following.

Reviewer #1 (Remarks to the Author):

This study by Shibata et al introduces an elegant and highly sophisticated new tool for research on CaMKII and synaptic plasticity: a photo-activatable paCaMKII. A long series of prominent previous pharmacological and genetic studies have established the necessity of CaMKII in LTP over the last 30 years. Using their novel tool, Shibata et al here for the first time convincingly test and demonstrate also sufficiency of CaMKII activation (using a realistic activation pattern). As LTP is thought to underly higher brain functions such as learning, memory, and cognition, these findings should be of high interest to a broad audience. This is further enhanced by the facts that the study design is beautiful, the experiments are rigorous, the scope is spanning from molecular mechanisms to in vivo analysis, and the writing is generally easily accessible.
While I have a rather lengthy list of comments, they are all very minor and do not require any additional experimentation.

Main comment:
The mechanism of autophosphorylation of paCaMKII deserves a little more discussion (because this is a new tool; because autophosphorylation is important for inducing plasticity; and because some quite interesting data are presented on it that may deserve some more attention). First of all, for endogenous CaMKII, T286 autophosphorylation occurs as a reaction between two subunits within a holoenzyme, and both subunits require binding of Ca2+/CaM. The data shown here indicate that photo-activation of paCaMKII can substitute for Ca2+/CaM not only on the subunit acting as kinase, but also on the subunit acting as substrate. This, in a way, is not unexpected, as the photoactivation should indeed make T286 accessible as substrate (but it would be good to explicitly mention this).

Thank you very much. We add the following lines.

Line 332
"Light illumination induces paCaMKII structural changes and autophosphorylation, suggesting that photoactivation of paCaMKII can substitute for Ca^{2+}/CaM binding to the subunit acting as kinase and substrate (i.e., T286 phosphorylation). This suggests that paCaMKII works similarly to endogenous CaMKII."

However, the data also indicate that paCaMKII co-assembles with endogenous CaMKII into heteromeric holoenzymes. In response to Ca2+-stimuli, all subunits get autophosphorylated; in response to light, only the paCaMKII subunits get autophosphorylated. This is great (and functionally important for this tool, as shown later), but it is slightly surprising that assembly into heteromeric holoenzyme does not seem to reduce the light-induced autophosphorylation of the paCaMKII (because addition of endogenous CaMKII should decrease the chance that paCaMKII assemble as neighbors to phosphorylate each other). This may be explained by insertion of the LOV2 domain inducing a "wider reach", i.e. beyond just the immediate neighboring subunit. But this would be worth discussing, perhaps also in context with estimates of the expression level estimates of endogenous CaMKII versus paCaMKII (to estimate how many paCaMKII subunits would be expected per holoenzyme).

In our biochemical experiment (Fig. 2d), we used dissociated neurons expressing paCaMKII. While the molar ratio of endogenous CaMKII and paCaMKII is 4:1 in "neurons in slices", the ratio of endogenous CaMKII/paCaMKII expression is 1:2 in "dissociated neurons". Please see right blotting in Fig. 2d. The band intensity of paCaMKII is higher than that of CaMKIIα. Thus, it makes us difficult to discuss about the things the reviewer suggested. However, we think it is important to discuss stoichiometry and activation of paCaMKII. Thus, we added the new paragraph in the discussion part (Line 321–342).

Further minor comments:
L38: change "leading to LTP" to "which can lead to LTP". (Overall the start of the introduction is nicely written to enable understanding by a wide non-expert audience; but this statement could be misinterpreted to indicate glutamate release always leads to LTP).

We fixed as following.

Line 35
"Post-synaptic receptors detect chemical transmitters, such as glutamate, which are released from pre-synapses and can lead to LTP."

L39: consider noting NMDARs as "NMDA-type glutamate receptors" (similarly as done a couple of lines down for AMPARs).

We fixed it (Line 38 and 42).

L46 and down: Many useful and relevant review articles on CaMKII are cited (including from Bayer and from Schulman), however, a more recent major review of CaMKII is missing (Bayer and Schulman, 2019 in Neuron).

Thank you very much. We cited it (Line 45, Ref. 8).

L60: consider rephrasing "researchers have developed".

Thank you very much. We rephrased it as following.

Line 57

"Recently, numerous genetically-encoded photoactivatable signaling proteins have been developed and used to study cellular functions[20], such as synaptic functions[21,22,23,24-26]. "

Fig1a-c is excellent for illustrating how the paCaMKII construct works. However, to the non-expert, it also suggests that CaMKII does exist to a significant extent also as monomeric subunit (which it does not). Thus, it might help to insert a panel that shows the CaMKII holoenzyme (likely as Fig1a or b).

Now, we added a figure of CaMKII/paCaMKII oligomer in Fig. 1d.

L92: "with the human beta-7 linker" is a bit misleading, I believe. To my understanding, the minor beta-7 isoform completely lacks a linker region. Thus, the paCaMKII construct has a LOV2 domain inserted and the linker region deleted. I believe there may actually be good rationale for deleting the linker region (which is relatively short in alpha anyway), such as that it might decrease the radius of how far the activated kinase domain can move away from the central hub (thereby compensating for the likely increase in radius introduced by insertion of the LOV2 domain). To make this point, inserting a panel with the holoenzyme in Fig1 may help (as also suggested above). For holoenzyme structure and radius considerations see also Myers et al 2017 in Nature Communications.

As the reviewer said we decided not to use "the human beta-7 linker". To make things clear, we add the amino acid sequence of paCaMKII as Supplementary Fig. 1, and we rewrote the following.

Line 89

"To compensate LOV2 insertion, we deleted a flexible linker region (315−344) which affects the configuretion and balance between active and inactive state[10,34,36], expecting that paCaMKII forms a similar configuration with endogenous CaMKII. We also expected that closed paCaMKII conformation in the dark would cause the regulatory domain to inhibit kinase activity, while light illumination would release the regulatory domain from the kinase domain, thereby activating paCaMKII (Figs. 1b–d, Supplementary Fig. 1)."

L108: It might be helpful to note here that the association domain mutations in the optimized paCaMKII do not affect holoenzyme formation (as indicated later in Fig1g).

Now, we added the following words.

Line 108
"These mutations minimize the response variability and improve the aggregation of the CaMKII FRET sensor, but do not affect the formation of oligomer[38]."

(L136: consider rewording the sentence.)

Thank you very much. We rephrased it as following.

Line 138
"Next, to test whether light selectively activates paCaMKII, we measured the autophosphorylation of paCaMKII, CaMKIIα, and CaMKIIβ upon blue light illumination. We observed that the light selectively induced the autophosphorylation of paCaMKII, but not CaMKIIα or CaMKIIβ (Fig. 2d)."

L140: Indicate duration of expression of the His-tagged paCaMKII here (especially as it is also unclear from the methods, which indicate infection a div 9-11 and harvest at div 12-15; so it could be 1 to 6 days for this experiment).

We replaced DIV 12–15 to ~72 hours to directory show the duration of the expression.

Line 958
"At DIV 9–11, primary neuronal cultures were infected with AAV-DJ particles at the concentration of 2.5 x 10^6 genome copies/ml. After ~72 hrs, the biochemical assay was carried out."

L141: reword, as there does not seem to be any evidence that phosphorylation at T286 is "critical" for activation of paCaMKII (even though it is later shown that it is indeed critical for the function of paCaMKII in plasticity).

We removed it ("and autophosphorylation of paCaMKII at T286 is critical for paCaMKII activation") as following.

Line 144
"In addition, pull-down assays with His-tagged paCaMKII confirmed that paCaMKII and CaMKIIα/β interact (Fig. 2e), indicating that paCaMKII and CaMKIIα/β form oligomers similar to endogenous CaMKII subunits."

L173: To my understanding, CaMKII interaction with L-type channels was described even before T- and P/Q-type interactions.

Thank you very much. Now, we added the following words with a citation (Ref. 42, McCarron et al. 1992, PMID: 1315424).

Line 177
"CaMKII is associated with L-, T-, and P/Q-type voltage-gated calcium channels[42-44]."

Fig.4d: Legend indicates paCaMKII activation for the sample traces (the measure one would want to see), but in the figure, "glutamate uncaging" is indicated instead.

Our description was little bit confusing. In Fig. 4d, we have replaced "stimulated" to "paCaMKII-activated spine". There, glutamate uncaging was just used to evoke EPSC. And the EPSC is compared before and after paCaMKII activation. And we also add the following sentence in the legend.

Line 739
"Note that, single-pulse of glutamate uncaging was used to evoke uEPSC, and the uEPSC before and after paCaMKII activation was compared in the same spine."

L257+262: "This difference suggests that other signaling pathways beside CaMKII pathway may be involved in the initial phase" and "These findings suggest that CaMKII-Cdc42 might be a major pathway for inducing sLTP." Consider rewriting in terms of sufficiency versus necessity of CaMKII. (CaMKII has been shown to be necessary for activation of both cdc42 and rhoA. These data indicated that it is also sufficient for activation of cdc42 but not rhoA). The current writing might be mis-interpreted to suggest that the CaMKII is involved only in activation of cdc42 but not rhoA. Similar for the discussion.

Thank you very much. We rephrased it as following.

Line 254
"This difference suggests that the initial phase of sLTP requires CaMKII and other signaling pathways."

Line 259
"These findings suggest that CaMKII activation is sufficient for triggering Cdc42 activation, but not RhoA activation."

We removed the following sentences from the discussion part.

"For example, we demonstrated that whereas glutamate activated both Cdc42 and RhoA, paCaMKII selectively activated Cdc42. The absence of RhoA activity may explain in part the difference in the early phase of sLTP, since RhoA may be important for triggering sLTP."

"Since we show that paCaMKII hardly activates RhoA, enhanced Cdc42 activation by this mechanism might involve Ras, Rac1, or other signaling molecules."

Fig. 7: title of the legend indicates in vitro and in vivo, but the data shown in this figure are exclusively in vivo imaging (the in vitro was shown in previous figures).

Thank you very much. We fixed it.

Line 822
"Fig. 8. paCaMKII activation triggers sLTP in cortical neurons *in vivo*."

L315: Consider rewriting, since in the current form, this is an overstatement. One can "separate" CaMKII from other Ca2+-activated processes by inhibition, to test necessity; the point is that this new approach allows direct activation to directly test also sufficiency. Perhaps consider this comparison also for the abstract, i.e. contrasting the long-known necessity of CaMKII in LTP with the here-shown sufficiency of CaMKII activation.

We rewrote it as following.

Line 27 in the abstract
"This optogenetic tool for dissecting the function of CaMKII activation (i.e., the sufficiency of CaMKII rather than necessity) and for manipulating synaptic plasticity will find many applications in neuroscience and other fields."

Line 310 in the discussion part
"The necessity of CaMKII for LTP has been extensively studied using various tools such as drugs, peptides, and siRNAs. However, it has been impossible to explore the sufficiency of CaMKII activation in a single dendritic spine. We describe a new genetically-encoded single molecule-type paCaMKII, which can be activated in single spines using 2-photon excitation. paCaMKII activation is sufficient to induce synaptic plasticity at the single-synapse level both *in vitro* and *in vivo*."

Reviewer #2 (Remarks to the Author):

In this manuscript Murakoshi et al. report the development of an optogenetic tool to control the Ca^{2+}/calmodulin-dependent protein kinase II (PACaMKII). The authors use the tool to study several aspects of synaptic plasticity involving CaMKII, including differential signaling in single vs. clustered spines and the requirement for Cdc42 activation in robust structural long-term potentiation. Both the tool and the biological studies are significant advances. However, concerns remain re both:

Major comments:

• Line 88: LOV's recovery rate can be tuned with point mutations. This is also true for other systems.

Thank you very much. We rephrased it as following.

Line 89

"Finally, the LOV2 works in a reversible manner (~40 s), similar to the others."

• Line 99-101: The authors provide very few details on one of the steps used to validate the optogenetic tool (2pFLIM-FRET) and the reader is referred to a supplementary figure. Because appropriate validation of the tool underpins all the biological conclusions, I suggest the authors expand more on this in the text and move some of the data in Fig S1 to the main text.

As the reviewer said we moved some of 2pFLIM-FRET data from supplementary fig to main figure (Fig. 1e-h). In addition, we inseted a new figure to show the basal fluorescence lifetime in Supplementary Fig. 2h, and expanded the validation part in the text.

Line 104

"The fluorescence lifetime change of mEGFP-paCaMKII-ShadowG in individual cells upon light illumination was variable ranging from 15 to 60 picoseconds (Supplementary Figs. 2a,i)."

Line 108

"These mutations minimize the response variability and improve the aggregation of the CaMKII FRET sensor, but do not affect the formation of oligomer[38]. The presence of these four mutations reduced light-dependent cell-to-cell variability (Supplementary Figs. 2a,b,i; *F*-test, $p = 2.675\times10^{-5}$, compared to the prototype) and suppressed kinase activity in both dark/light conditions (Supplementary Fig. 3b)."

• Fig S1 j): I539E (lit mutant) has a significantly lower lifetime than wild type PaCaMKII. Shouldn't this mutant have identical or higher lifetime than wild type PaCaMKII?

I539E mutant have higher fluorescence lifetime. We showed a distribution of basal (before light) fluorescence lifetime in supplementary fig. 2h. I539E mutant exhibited longer fluorescence lifetime than wildtype paCaMKII, suggesting that the significant fraction of I539E mutant takes open form.

Now, we added the following words.

Line 130
"Introducing a constitutively-open-form I539E mutation[27] in the Jα helix induced a relatively high basal fluorescence lifetime, most likely due to the open form of the mutant (Supplementary Fig. 2h)."

• Line 112 and Supplementary Fig. 2: "(…) suppressed background kinase activity in the dark condition (…)". This is not clear when comparing prototype with prototype (4m); both light and dark bars seem to decrease and the ratio seems identical.

The introduction of 4m reduces the activity in both dark/light, So, we rephrased it as following.

Line 110
"The presence of these four mutations reduced light-dependent cell-to-cell variability (Supplementary Figs. 2a,b,i; *F*-test, $p = 2.675\times10^{-5}$, compared to the prototype) and suppressed kinase activity in both dark/light conditions (Supplementary Fig. 3b)."

In Figure S2, it is unclear what "divided by EGFP" means when image a) only displays phosphorylated and total mEGFP-PACaMKII.

We fixed y-label as "Fold change of pT286 CaMKII" in Fig. S3b. And we add the explanation in the legend.

Supplementary Figure 3
"(b) Quantification of (a). The band intensity of lane #1 was normalized for comparison, and the normalized band intensities of p286 were divided by those of total. Error bars indicate SEM for three independent experiments. The number of samples (n) at each point is three."

How do the authors justify the clear difference in prototype and prototype (4m) being absent in Fig S1 j, where no statistical difference is found for the FLIM assay, and what is described as a "reduced light-dependent response variability" which does not seem to hold true in the blotting data for the S279K mutation?

Here, I would like to say that prototype (4m) reduces “the cell-to-cell” response variability. So, the blotting data is not relevant to it. To show the difference of the response variability, we have carried out F-test in Fig. 2i and rephrased as the following.

Line 110
“The presence of these four mutations reduced light-dependent cell-to-cell variability (Supplementary Figs. 2a,b,i; *F*-test, $p = 2.675\times10^{-5}$, compared to the prototype) and suppressed kinase activity in both dark/light conditions (Supplementary Fig. 3b).”

• Fig 1d): no loading controls are provided, preventing direct comparison of number of cells and expression levels.
I would suggest the authors include the full, uncropped blots as Supplementary Material for these and other Western Blotting data.

We included loading controls in Fig. 2a and b, and provided uncropped blots for all data as supplementary material (Supplementary Fig. 7).

And we explicitly stated that the band intensity was divied by total in the legend of Fig. 2.

Line 659
“(**c**) Quantification of (a,b). The number of samples (n) at each point is 3–8. The band intensity of before light was used for normalization. The band intensities of p286 were divided by those of total.”

• Lines 140-143, Figure 1F: Doesn’t the data from NMDA stimulation in the dark show that this tool still responds to upstream signaling, i.e. is not solely controlled by light? This indicates that expression perturbs resting CaMKII signaling, so at the very least expression level should be carefully controlled and effects in the dark should be gauged as a function of expression. This should be discussed in the text.

We added the following words.

Line 325
“One concern is the effect of CaMKII overexpression. For example, since paCaMKII is activated by NMDA in a Ca^{2+}-dependent manner, Ca^{2+}-dependent paCaMKII activity may disturb resting CaMKII signaling. However, this effect should be limited because the paCaMKII expression level in our experiments relatively low (~15 μM) compared to endogenous CaMKII (~60 μM in proximal dendrites)[56]. Since the holoenzyme is expected to consist of 4:1 endogenous CaMKII to paCaMKII, it may not have a profound effect on the signaling.”

• Lines 169-170 and Fig2 h and i: The authors’ method to estimate the concentration of PaCaMKII is problematic. Because of the p2a ribosomal skip sequence, expression of tdTomato and PaCaMKII will be identical but degradation

rates can differ massively; the concentration of the two species can't simply be assumed to be identical. Furthermore, the linear fits are not useful on what seems to be a more stochastic than trended distribution. Lastly, in the Materials and Methods pertaining to this experiment, I would suggest the authors describe approximate volume for apical dendrites as opposed to "thick apical dendrites".

We agree to the reviewer's comment. Certainly, the degradation rate or translation efficiency might be different between tdTomate and paCaMKII. So, in supplementary fig. S4, we estimated the expression level of tdTomato and paCaMKII as tdTomato-P2A-paCaMKII is expressed in neurons, and found that the expression ratio of tdTomato and paCaMKII was 2:1.

Now, we added the following words.

Line 701
"The tdTomato expression was measured by comparing the fluorescence intensity of tdTomato in proximal dendrites and a known concentration of purified tdTomato under a 2-photon microscope. Because the ratio of tdTomato and paCaMKII expression is 2:1 (See Supplementary Fig. 4), the scale for paCaMKII concentration is presented as well."

Certainly, the liner fits mean nothing here. We removed the linear fits from the figure (Figs. 3h,i).

We inserted "3–6 µm in diameter" as the following.

Line 938
"The concentration of tdTomato in neurons was estimated by measuring the fluorescence intensity of tdTomato in thick apical dendrites (3–6 µm in diameter) relative to that of purified tdTomato (10 µM) under a 2-photon microscope."

• Figure 3a: Images provided for GCaMP imaging are particularly noisy when compared to all other images in the document. Do the authors have an explanation for this?

To measure the calcium ion influx into spines, the relatively high temporal resolution is required for the measurement. The images in Fig. 4a were slightly noisy because they were acquired at 15.6 Hz (64 ms/frame) with "single scan" as described in the figure caption, while the other images were the average or integration of 12–24 images. We inserted these explanation as the following.

Line 711
"Images were slightly noisy because they were acquired at a high temporal resolution (15.6 Hz)."

• Figure 3b: This panel would benefit from control spine measurements near the PA and glutamate uncaging as done elsewhere in Figure 2.

In fig. 4b, to acquire the images at a high rate (15.6 Hz), the region of images is limited. Thus, most of our image data used in figure 4 do not have adjacent spines. In addition, the aim of figure 4 is to test if the calcium influx occurs upon glutamate/paCaMKII stimulation or not. Since the specificity of sLTP is provided in other figures, we think that the data for the adjacent spine is not required.

• Lines 292-298 and Fig 6: Authors employ a single shRNA to knockdown Cdc42; I would suggest validation with a second shRNA to confirm these results, particularly because the control seems to have an effect when compared to knockdown and rescue. This could also stem from a lack of the wild type data to directly compare these effects.

Thank you very much. As the reviewer says, the control (shCtrl in Fig. 7a) seems to affect spine enlargement, i.e., volume change looks smaller than that in Fig.2b. There may be two possibilities. One is that our control shRNA suppresses the spine enlargement due to the off-target effect. The other is the condition of slice culture due to seasonal differences and so on. To judge if our shCtrl affects spine enlargement, we compared the spine volume change upon paCaMKII activation in the presence or absence of shCtrl (see the following figs.) using the same batch of culture slices. And we found that there is no significant difference between the two experiments, suggesting that our shCtrl does not affect spine enlargement. Therefore, we think the moderate level of spine enlargement in Fig. 7a is just due to the variability of slice batch or seasonal difference. Indeed, the spine volume change during 4-6 min is variable, i.e., delta volume (%) during 4-6 min is 200% in Fig.2b, 100% in Fig. 3, 180% in Fig. 5e, 180% in Fig. S4. Since we have done our experiment with the same batch of slices for each figure, the moderate spine volume is not problematic. For shCdc42 experiments in Fig. 7, the specificity of shCdc42 was confirmed by doing the rescue experiment with shRNA resistant Cdc42. Thus, we think the experiments with a second shRNA are not required at the current stage.

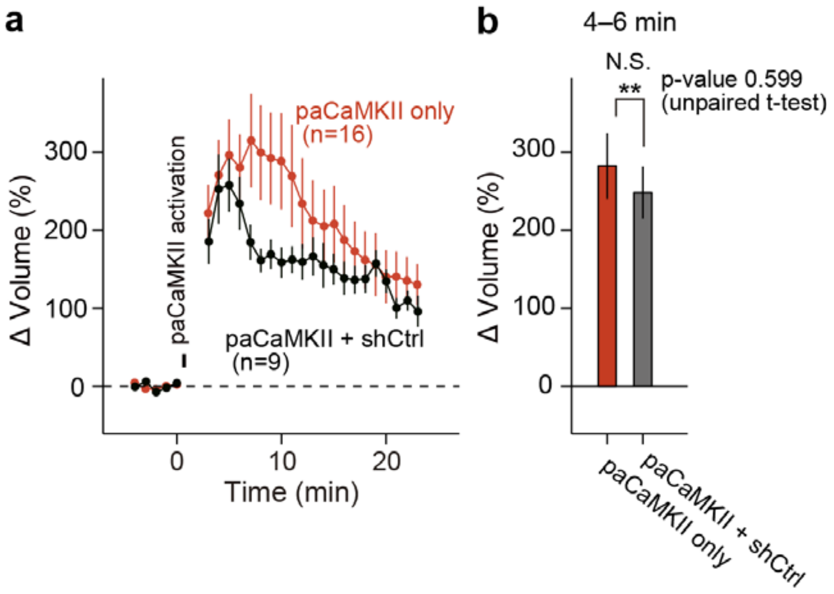

• Two photon considerations: I have several concerns regarding these experiments:

o Lines 84-88: Comparison of two-photon absorption cross section values with FAD is only relevant for the CRY2 system and does not address others. References provided refer to free FMN (not in a protein). Reference 70 would be more appropriate here; providing actual values would be beneficial too.

Thank you very much. We have replaced the reference to Ref. 32(Homans et al. 2018) (previously Ref. 70). And we rewrote as the following.

Line 80
"Furthermore, to our knowledge, while the 2-photon cross sections of Dronpa, PhyB, and UVR8 have not been determined, the light-absorbing cofactor of LOV2, flavin mononucleotide (FMN), has a relatively large 2-photon cross-section (0.5–0.9 GM in 800–900 nm)[32]. It is larger than that of flavin adenine dinucleotide (FAD), the light-absorbing cofactor for CRY2 (0.02–0.04 GM in 800–900 nm)[33]. Finally, the LOV2 works in a reversible manner (~40 s), similar to the others."

o Lines 337-339: authors cite values of ~0.2 GM at 800-900 nm for iLOV, yet the reference provides values ranging from ~1 to that value. The reference provided for MNI-glutamate TPA cross section only provides a single value at a single wavelength, which complicates this comparison. Do the authors have a reference for a full TPA spectrum?

We found a full TPA spectrum of MNI-glutamate (Fig. 8 in PMID: 29497727) although it is not in GM value.

We cited the above paper (Ref. 59) and rephrased as the following.

Line 353
"Fourth, since the 2-photon cross-section of LOV2 (0.5–0.9 GM in 800–900 nm[32]) is higher than that of MNI-glutamate (0.06 GM at 720 nm, the most widely used wavelength for efficient photolysis)[58,59],…."

o Cross-photoactivation by label and biosensor imaging: Even though iLOV TPA seems to mostly drop close to zero at >950 nm, studies for closely related FMN-binding proteins (e.g. Nanna et al. 2014) show higher TPA cross section values. Determining particularly low TPA cross section values is prone to large variations. Furthermore, a small but noticeable effect is visible on volume change and binding fraction in control spines (Fig 2 b), Fig 5 c and e). This should at least be addressed in the text; ideally the authors should show that wavelengths of ≥1000 nm do not photoactivate PaCaMKII, extend their period before photoactivation, and/or perform a control experiment where cells

containing PaCaMKII and labels (tdTomato and sensors) are imaged without photoactivation pulses.

As the reviewer said we carried out a control experiment (no light stimulation) with neurons expressing paCaMKII and Cdc42 FRET sensor (Fig. 6c-f, paC no light).

Line 764
"A control experiment with no light stimulation is also plotted (paC no light). Even without light stimulation, slight increase in binding fraction (~1%) and volume change (~10%) was observed. This may be due to the cross-photoactivation of paCaMKII by 1010 nm imaging laser during observation.

We also mentioned about the possibility of cross activation in the following.

Line 158
"Upon paCaMKII activation, we observed a slight increase in adjacent spine volume (Figs. 3b,d). This might be due to paCaMKII cross-photoactivation by the 1000 nm imaging laser during observation."

Other comments:

• Line 82: The exact size depends on which LOV protein is chosen, I would suggest authors change this to ~140 a.a.

Thank you very much. We changed it.

Line 78
"Second, LOV2-Jα, is ~140 amino acids (a.a.), which is smaller than most other sensors (i.e., CRY2/CIBN 498/170 a.a., Dronpa 257 a.a., PhyB 908 a.a., and UVR8 124 a.a.)."

• Line 389: I believe the authors meant "directly identify" and not "directory identifies".

We fixed it.

Line 394
"we should be able to directly identify the various downstream signaling molecules of CaMKII"

• Fig1: I would suggest the authors change their naming of main experiment "Ctrl" to "Stim" as used elsewhere, so the reader does not think it is a control.

Thank you very much. In fig. 3, we changed "Ctrl" to "Stim".

• Materials and Methods: “Note that equation (1) can be applied to spherical structure Since the dendrite cannot be assumed as spherical, Rdendrite_surface is an approximate calculation.” I would suggest the authors reword this phrase.

We rephrased it as following.

Line 1103

“Since the dendrites are not spherical structure, $R_{dendrite\_surface}$ is an approximate calculation.”

• Can the authors explain why they started with mScarlet but switched to tdTomato for most of the experiments?

It was our mistake. We did not use mScarlet. In the manuscript, we described that mScarlet-P2A-paCaMKII was used for biochemical assay for timecourse experiment. But, what we actually used for the assay is Flag-His×6-paCaMKII. We corrected this point in the current MS.

Line 655

“Dissociated hippocampal neurons were infected with AAV-DJ encoding Flag-His×6-paCaMKII or mutant under CaMKII promotor were illuminated with blue light for 3 min.”

• I would suggest significance values be provided in each image where statistical analysis is performed (and what test performed).

We have provided significance values and the test-type in each figure caption.

REVIEWERS' COMMENTS

Reviewer #1 (Remarks to the Author):

The authors have sufficiently addressed all of my initial concerns.

Reviewer #2 (Remarks to the Author):

The authors have done a good job of addressing all my suggestions and I am fine with the manuscript in its current form. There is one minor point that I do not understand. The half life for return to the dark state of LOV is approximately 27s, and mutations can be used to shorten this to about 5 seconds. In their arguments for using LOV2 the authors mention, as a LOV2 advantage, a point that is often erroneously used to argue that LOV2 is not so useful. LOV2 is sometimes said to have a long half life for return to the dark state. One reference that refers to studies of mutations which can be used to change the lifetime is Wang et al. 2016PMC5137945. I would suggest simply leaving out the (-40s) in the sentence below, or using an accurate value of half life.

“Finally, the LOV2 works in a reversible manner (-40s) similar to the others.”

Response to reviewers

We would like to thank the reviewers for accepting our manuscript.

Reviewer #1 (Remarks to the Author):

The authors have sufficiently addressed all of my initial concerns.

Thank you very much.

Reviewer #2 (Remarks to the Author):

The authors have done a good job of addressing all my suggestions and I am fine with the manuscript in its current form. There is one minor point that I do not understand. The half life for return to the dark state of LOV is approximately 27s, and mutations can be used to shorten this to about 5 seconds. In their arguments for using LOV2 the authors mention, as a LOV2 advantage, a point that is often erroneously used to argue that LOV2 is not so useful. LOV2 is sometimes said to have a long half life for return to the dark state. One reference that refers to studies of mutations which can be used to change the lifetime is Wang et al. 2016PMC5137945. I would suggest simply leaving out the (-40s) in the sentence below, or using an accurate value of half life.

Thank you very much. We have left out the (-40 s).
